# On the Mathematical Relationship Between Contextual Probability and N400 Amplitude

**DOI:** 10.1162/opmi_a_00150

**Published:** 2024-06-28

**Authors:** James A. Michaelov, Benjamin K. Bergen

**Affiliations:** Department of Cognitive Science, University of California San Diego

**Keywords:** surprisal, information theory, event-related brain potentials, N400, neural language models, natural language processing, language comprehension, human language processing, psycholinguistics

## Abstract

Accounts of human language comprehension propose different mathematical relationships between the contextual probability of a word and how difficult it is to process, including linear, logarithmic, and super-logarithmic ones. However, the empirical evidence favoring any of these over the others is mixed, appearing to vary depending on the index of processing difficulty used and the approach taken to calculate contextual probability. To help disentangle these results, we focus on the mathematical relationship between corpus-derived contextual probability and the N400, a neural index of processing difficulty. Specifically, we use 37 contemporary transformer language models to calculate the contextual probability of stimuli from 6 experimental studies of the N400, and test whether N400 amplitude is best predicted by a linear, logarithmic, super-logarithmic, or sub-logarithmic transformation of the probabilities calculated using these language models, as well as combinations of these transformed metrics. We replicate the finding that on some datasets, a combination of linearly and logarithmically-transformed probability can predict N400 amplitude better than either metric alone. In addition, we find that overall, the best single predictor of N400 amplitude is sub-logarithmically-transformed probability, which for almost all language models and datasets explains all the variance in N400 amplitude otherwise explained by the linear and logarithmic transformations. This is a novel finding that is not predicted by any current theoretical accounts, and thus one that we argue is likely to play an important role in increasing our understanding of how the statistical regularities of language impact language comprehension.

## INTRODUCTION

The N400 (Kutas & Hillyard, 1980, 1984) is a negative component of the event-related brain potential that peaks around 400 ms after the presentation of stimulus and is associated with lexical and semantic processing difficulty (Aurnhammer & Frank, 2019b; Brouwer et al., 2021; Federmeier, 2021; Kutas et al., 2006; Thornhill & Van Petten, 2012). Specifically, the amplitude of the N400 response to a stimulus has been found to be large by default, and is reduced (becomes less negative) when neural representations of the stimulus are preactivated—that is, activated by the preceding context (DeLong & Kutas, 2020; DeLong et al., 2014; Federmeier, 2021; Kuperberg et al., 2020; Van Petten & Luka, 2012). In the language domain, it is by now well-established that the amplitude of the N400 response to a word is highly correlated with the word’s contextual probability, whether this is operationalized based on human judgements (Kutas & Hillyard, 1984; for reviews see DeLong et al., 2014; Federmeier, 2021; Kuperberg et al., 2020; Kutas & Federmeier, 2011; Kutas et al., 2011; Van Petten & Luka, 2012) or the statistics of language (Aurnhammer & Frank, 2019b; Frank et al., 2015; Merkx & Frank, 2021; Michaelov et al., 2022, 2024; Parviz et al., 2011; Szewczyk & Federmeier, 2022).

The canonical way to operationalize contextual probability is using the cloze task, where the cloze probability of a word in a given context is the proportion of participants in a norming study who fill in the gap in a sentence with that word (Taylor, 1953, 1957). Since the relationship between cloze probability and the N400 was first discovered (Kutas & Hillyard, 1984), the finding that the two are correlated has been replicated numerous times (see, e.g., DeLong et al., 2014; Federmeier, 2021; Kuperberg et al., 2020; Kutas & Federmeier, 2011; Kutas & Van Petten, 1994; Van Petten & Luka, 2012), with some studies finding as high a degree of correlation between the two as *r* = 0.9 (Kutas & Federmeier, 2011; Kutas & Van Petten, 1994).

A more recent approach uses the contextual probabilities calculated by computational language models. Language models are systems designed to calculate the probability of a word given a context based on the statistics of language (Jurafsky & Martin, 2024), and like cloze, these probabilities have also been found to be highly correlated with N400 amplitude (Aurnhammer & Frank, 2019a, 2019b; Frank et al., 2015; Merkx & Frank, 2021; Michaelov et al., 2021, 2022, 2024; Parviz et al., 2011; Szewczyk & Federmeier, 2022; Yan & Jaeger, 2020). In addition to sometimes displaying a closer fit to the N400 data than cloze (Michaelov et al., 2022, 2024), these language model probabilities have a higher degree of explanatory power from an information-processing perspective—they allow researchers to specifically test to what extent the statistics of language may influence the preactivation underlying the N400 response.

The precise mathematical relationship between these language-model-derived word probabilities and the amplitude of the N400 responses elicited by the same words in humans is of prime theoretical importance because it can in principle adjudicate among mechanistic accounts of language processing and of the N400 specifically. But the nature of that relationship is currently unknown, and we focus on this key question in the present study.

In the related literature on reading time, another type of measure which is also thought to reflect processing difficulty and to be impacted by contextual probability, the question has been studied extensively, and a range of possibilities have been proposed and tested. Most notably, the relationship between word probability and processing difficulty has been argued to be linear (Brothers & Kuperberg, 2021), logarithmic (Shain et al., 2024; Smith & Levy, 2013; Wilcox et al., 2023), or an exponential transformation of the logarithmically-transformed values (Hoover et al., 2023; Levy & Jaeger, 2006; Meister et al., 2021). The results from large-scale meta-analyses of reading time suggest a linear relationship between cloze and processing difficulty (Brothers & Kuperberg, 2021), but for corpus-derived probabilities (i.e., calculated using language models), there appears to be evidence for both a logarithmic (Shain et al., 2024; Smith & Levy, 2013) and a ‘super-logarithmic’ relationship (Hoover et al., 2023; Meister et al., 2021), the latter being a term used to describe a super-linear relationship between log-probability and processing difficulty (see Levy & Jaeger, 2006; Shain et al., 2024; Smith & Levy, 2013).

In contrast to reading time, there has been comparatively little work investigating the mathematical nature of the relationship between lexical probability and N400 amplitude, and the results are far from conclusive. Of the three studies that we are aware of that have looked at the relationship between cloze probability and the N400, two (Aurnhammer et al., 2021; Michaelov et al., 2022) found log-transformed cloze probability to correspond slightly more closely to N400 amplitude, while the other (Szewczyk & Federmeier, 2022) found the reverse. Thus far, only two studies have investigated the relationship between statistical (i.e., corpus-derived) lexical probability and N400 amplitude. Yan and Jaeger (2020), using the probabilities derived from a hybrid model based on a mixture of a 5-gram model and ‘skip bi-gram’ (see Frank & Willems, 2017), find that surprisal (negative log-probability) better predicts N400 amplitude than un-transformed probability does. This finding is also replicated by Szewczyk and Federmeier (2022), who find that overall, surprisal derived from the GPT-2 language model (Radford et al., 2019) out-performs un-transformed GPT-2 probability as a predictor of N400 amplitude. However, Szewczyk and Federmeier (2022) also find that GPT-2 probability explains variance in N400 amplitude above and beyond that explained by GPT-2 surprisal, and may be a better predictor for more expected words (cloze > 0.05). Thus, the question of the mathematical relationship between the language-model-derived probability of a word and the amplitude of the N400 response to the word is still far from resolved.

In order to address this, we expand upon previous work in several ways. First, the question of the mathematical relationship between language-model-derived probability and N400 amplitude has only been tested for two language models; we analyze data from 37 contemporary transformer language models. Additionally, previous work has only compared the extent to which probability and surprisal predict N400 amplitude. In the current study, we also investigate a range of sub-logarithmic (surprisal to a power < 1) and super-logarithmic (surprisal to a power > 1) relationships in the same vein as some previous work on reading time (Meister et al., 2021; Shain et al., 2024). We use these approaches to re-analyze the five datasets used in the Szewczyk and Federmeier (2022) study (Federmeier et al., 2007; Hubbard et al., 2019; Szewczyk & Federmeier, 2022; Szewczyk et al., 2022; Wlotko & Federmeier, 2012), along with data from a large-scale study carried out by Nieuwland et al. (2018).

## THEORETICAL ACCOUNTS AND THEIR MATHEMATICAL FORMULATIONS

Theoretical accounts of the N400 and processing difficulty in general differ in the mathematical relationships that they propose may hold between contextual probability and processing difficulty. In this section, we describe a range of such theoretical accounts and the mathematical relationships they propose.

### Contextual Probability

There is a long history of studies using cloze probability as a predictor of N400 amplitude (since Kutas & Hillyard, 1984) or of behavioral measures such as reading time (since Fischler & Bloom, 1979). As Brothers and Kuperberg (2021) note, using cloze probability as a predictor of N400 amplitude implicitly assumes a linear relationship between contextual probability and processing difficulty. Brothers and Kuperberg (2021) use this previous work as a basis upon which to build a theoretical framework supporting such a linear relationship, which they term the *proportional preactivation* account.

Mechanistically, processing difficulty as described by the proportional preactivation account aligns with the majority of the contemporary accounts of the N400. As a basic principle, processing difficulty reflects the effort required to activate neural representations driven by the stimulus encountered. Difficulty is reduced by the extent to which these representations were preactivated—that is, already activated at the time that the stimulus was encountered (Federmeier, 2021; Kutas & Federmeier, 2011). Under the proportional preactivation account (as in DeLong & Kutas, 2020; DeLong et al., 2005, 2014; Kuperberg et al., 2020; Kutas et al., 2011; Van Petten & Luka, 2012), preactivation is largely driven by prediction based on the preceding context. Crucially, under the proportional preactivation account account, words are preactivated in direct proportion to their contextual probabilities. And so, given that processing difficulty reflects the difference between the extent to which a word is preactivated and its full activation state, we should expect probability to be linearly related to processing difficulty (Brothers & Kuperberg, 2021).

### Distribution Update

Under the proportional preactivation and other contextual probability accounts, processing difficulty arises from the extent to which the stimulus itself is predictable based on its context. An alternative idea is that processing difficulty also reflects the probability of alternatives and the difficulty in disconfirming them. This idea forms the basis of accounts (e.g., Frank et al., 2013; Hale, 2001; Levy, 2008; Smith & Levy, 2013) which we term *distribution update* accounts, and which are often grouped under the category of *surprisal theory* because they posit a linear relationship between processing difficulty and *surprisal*, the negative log-probability of a word given its preceding context.

Such accounts vary in their specific details and formalizations, but at their core they share the idea that as humans comprehend linguistic input, we allocate our neurocognitive resources among different possible parses or interpretations of the current input (Hale, 2001; Levy, 2008), or among different possible next words in the utterance (Aurnhammer & Frank, 2019b; Frank et al., 2013). Specifically, resources are divided such that more likely candidates are allocated a larger amount than less likely candidates, in proportion to their probability. Processing difficulty, then, is the effort required to update the distribution over possible candidates after encountering a given stimulus. Notably, whether this is directly formalized as surprisal (Frank et al., 2013; Hale, 2001) or as the Kullback-Leibler divergence (Kullback & Leibler, 1951) between the probability distribution before and after a word is encountered (Aurnhammer & Frank, 2019b; Levy, 2008), it can be shown formally that this effort can mathematically be described as surprisal.

In this study, we specifically focus on the formulation of the distribution update account presented (among others accounts) by Aurnhammer and Frank (2019b). Under this account, the state of the language comprehension system before encountering a lexical stimulus can be modeled as a probability distribution over possible next words given the preceding context, and the state after can be modeled as the true probability distribution—a distribution where the actual stimulus has a probability of 1 and all other words have a probability of 0. Processing difficulty under this account is precisely the effort required to ‘collapse’ the predicted probability distribution to the true probability distribution after encountering a word. This effort can be modeled as the Kullback-Leibler divergence between the two probability distributions, which, as Aurnhammer and Frank (2019b) note, is mathematically equivalent to surprisal.

### Composite Processing Difficulty of Sub-word Features

The second, related family of logarithmic accounts posits that the relationship between contextual probability and processing difficulty arises not from a direct relationship between the contextual probability of a word and processing difficulty, but rather between sub-components of the word and processing difficulty (Smith & Levy, 2008, 2013). A key account of this kind is the ‘highly incremental’ account presented by Smith and Levy (2013). The account proposes that rather than occurring at the word level, the effect of probability on lexical processing difficulty might instead arise at the sub-word level, that is, in the processing of each consecutive sub-word fragment of a word. Crucially, the probability of each consecutive fragment of a word impacts word probability multiplicatively (i.e., the probability of a word *w*_*i*_ made up of chunks *c*_1_ … *c*_*k*_ is given by *p*(*w*_*i*_) = *p*(*c*_1_) × … × *p*(*c*_*k*_)), but if each chunk is processed sequentially, its impact on reading time *t* is additive (i.e., *t*(*w*_*i*_) = *t*(*c*_1_) + … + *t*(*c*_*k*_)). As Smith and Levy (2013) prove and demonstrate with examples, as *k* increases, any function *f* that relates *p*(*w*_*i*_) to *t*(*w*_*i*_) tends toward a linear function of *log*(*p*(*w*_*i*_)). Thus, the account argues for a logarithmic relationship between contextual probability and processing difficulty.

While the account makes sense in the context of reading time, it is less clear whether it can account for the N400. Specifically, the highly incremental account relies on additional time taken for each sub-word chunk processed. When measuring processing difficulty using the N400, on the other hand, the focus is on amplitude over a given time period (generally 300–500 ms after stimulus presentation), and it is not straightforward to imagine a mechanism whereby the difficulty in processing of incremental sub-word fragments would increase the amplitude in the same fixed-time period. Szewczyk and Federmeier (2022), however, propose an alternative account along the same lines that focuses on semantic features rather than sub-word fragments. Under this account, the probability of a word in a given context is the product of the probability of each of its semantic features, but the effect of the probability of each feature on N400 amplitude is linear. Following Smith and Levy (2013), therefore, if there are a sufficient number of semantic features associated with a given word—and it is difficult to imagine cases where words are not associated with many semantic features—we should expect N00 amplitude to be logarithmically related to contextual probability (Szewczyk & Federmeier, 2022).

While it is in principle possible to view the reading-time variants of this account (Smith & Levy, 2008, 2013) as identifying possible mechanisms by which distribution update accounts such as those provided by Hale (2001) or Levy (2008) could occur, this is not the case with N400-focused account. Crucially, under the formulation of the distribution update account provided by Aurnhammer and Frank (2019b), surprisal indexes lexical predictions, while Szewczyk and Federmeier (2022) argue instead that lexical predictions lead to differences in N400 amplitude that are linearly related to contextual probability.

### Uniform Information Density

In addition to linear and logarithmic relationships, it is also possible that processing difficulty is non-linearly related to log-probability. The main argument for this comes from considering how information is distributed throughout a sentence. Intuitively, one would expect that sentences where all the information is concentrated into a small number of words would be harder to comprehend than those where information is more evenly spread out (Levy, 2005), and it is possible that this may result in a pressure towards more uniform information density in sentence production (Levy & Jaeger, 2006; Meister et al., 2021; Shain et al., 2024; Smith & Levy, 2013). Specifically, Levy and Jaeger (2006)^1^ demonstrate mathematically that if there is a super-logarithmic relationship between lexical probability and processing difficulty (i.e., difficulty (−*log*(*p*))^*k*^ where *k* > 1), then uniform information density minimizes the effort required to process a whole utterance.

While there is a substantial body of both theoretical and empirical work arguing in favor of uniform information density in general (see, e.g., Aylett & Turk, 2004; Clark et al., 2023; Coupé et al., 2019; Fenk & Fenk-Oczlon, 1980; Genzel & Charniak, 2002; Maurits et al., 2010), the question of whether it arises from comprehender-oriented principles (i.e., a form of audience design) is still an open question. Thus, a super-logarithmic relationship between contextual probability and processing difficulty could help explain why language users produce the utterances that they do.

### Multiple Sub-components

In addition to accounts proposing one specific mathematical relationship between contextual probability and processing difficulty, some have proposed that a combination of relationships holds between the two. Szewczyk and Federmeier (2022) propose, for example, that the N400 is related to contextual probability both logarithmically and linearly. The account provided by Szewczyk and Federmeier (2022) follows from accounts of the N400 under which the response is posited to generally reflect the overlap between the semantic features of the stimulus and its context, and to only under some conditions (for example, when more attention is paid to stimuli) reflect explicit lexical prediction (see, e.g., Federmeier, 2021; Lau et al., 2013). Szewczyk and Federmeier (2022) specifically argue that the logarithmic relationship reflects the effect of semantic feature overlap (proposing the aforementioned account of logarithmic composite processing difficulty based on sub-word semantic features), and that the linear relationship reflects the effect of lexical prediction (following contextual probability accounts like the proportional preactivation account). While they do not provide direct evidence for the correspondence between these components and the proposed mechanisms indexed, Szewczyk and Federmeier (2022) do provide direct evidence of potentially separable linear and logarithmic effects. Specifically, Szewczyk and Federmeier (2022) find that the linear effect is only significant above and beyond the logarithmic for expected items (cloze > 5%) when analyzing the whole N400 time window (300–500 ms); and is only significant when predicting the N400 response to all tokens when analyzing the first half of the time window (300–400 ms).

## ANALYSIS 1: POWERS OF SURPRISAL

### Introduction

The aim of the present study is to investigate whether the relationship between contextual probability and N400 amplitude is linear, logarithmic, super-logarithmic, or sub-logarithmic. In Analyses 2 and 3 below we will compare how well each of these transformations of probability predict N400 amplitude. This first analysis sets the stage by identifying the super- or sub-logarithmic transformation of probability that best correlates with N400 amplitude (in order to subsequently compare this with probability and surprisal).

As previously discussed, the current evidence from reading time suggests either a logarithmic (Shain et al., 2024; Smith & Levy, 2013; Wilcox et al., 2023) or super-logarithmic relationship (Hoover et al., 2023; Meister et al., 2021) between contextual probability and processing difficulty. However, it is also in principle possible that there is a sub-logarithmic relationship, and as this type of analysis has never been carried out for the N400, we account for both possibilities. Specifically, we calculate all surprisal^*k*^, where *k* covers all 0.1 increments between 0.1 and 2 (inclusive), as well as −1,−0.5, and −0.1, for comparison. We then test how well each of these predicts N400 amplitude.

### Method

#### Language Models.

Recent research shows that among contemporary language model architectures, N400 amplitude is best predicted by transformers (Merkx & Frank, 2021; Michaelov et al., 2022). We therefore restricted our analysis to contemporary transformer language models made available through the *transformers* (Wolf et al., 2020) Python (Van Rossum & Drake, 2009) package. We further restrict our analyses to only include autoregressive (unidirectional) transformer language models, as the best-performing model in Michaelov et al. (2022) was of this type, and because they produce well-defined probabilities for critical words made up of multiple tokens, allowing us to calculate surprisal for any word. These included 37 models of the GPT-2 (Radford et al., 2019), GPT-Neo (Black et al., 2021), GPT-J (Wang & Komatsuzaki, 2021), Pythia (Biderman et al., 2023), OPT (Zhang et al., 2022), XGLM (Lin et al., 2021), and BLOOM (BigScience, 2022) architectures, as well as DistilGPT2, a ‘distilled’ form of GPT-2 (see Sanh et al., 2020).

#### Stimuli and N400 Data.

We use the stimuli and experimental data from 5 previously-published N400 studies (Federmeier et al., 2007; Hubbard et al., 2019; Nieuwland et al., 2018; Szewczyk et al., 2022; Wlotko & Federmeier, 2012), and one unpublished dataset released as part of a recent meta-analysis (Szewczyk & Federmeier, 2022).

In the 5 datasets (Federmeier et al., 2007; Hubbard et al., 2019; Szewczyk & Federmeier, 2022; Szewczyk et al., 2022; Wlotko & Federmeier, 2012) preprocessed and released by Szewczyk and Federmeier (2022), N400 amplitude was operationalized as the mean voltage from four centro-parietal electrodes (MiCe, MiPa, LMCe, RMCe), and the mean was taken over the 300–500 ms time window. In contrast to much of the work on the N400, in this dataset, N400 amplitudes are not corrected using a baseline amplitude; instead, the −100–0 ms mean amplitude baseline is included as a covariate in analysis (see discussion in Szewczyk & Federmeier, 2022). The details of these datasets are presented in Table 1. The stimuli from the study by Federmeier et al. (2007) follow a 2 × 2 design, with sentences either having a high or low constraint, and the N400 being recorded from either an expected (highest-cloze) or unexpected (low-cloze) continuation. The stimuli from the other studies were generally selected from Federmeier et al. (2007), with Wlotko and Federmeier (2012) including additional sentences with critical words that varied more continuously in terms of their cloze probability, and Szewczyk et al. (2022) adding adjectives that either reduced or increased the cloze probability of critical items.

**Table 1.** Details of all datasets analyzed.

**Dataset**	**Stimuli**	**Participants**	**Total trials**
Federmeier et al. (2007)	564	32	7856
Wlotko and Federmeier (2012)	300	16	4440
Hubbard et al. (2019)	192	32	5705
Szewczyk et al. (2022)	672	32	4939
Szewczyk and Federmeier (2022)	600	26	4822
Nieuwland et al. (2018)	160	334	25978

The remaining dataset is a large-scale study carried out by Nieuwland et al. (2018). We take the subset of the data corresponding to N400 amplitudes elicited by nouns. We use the preprocessed data provided by Nieuwland et al. (2018), who operationalize N400 amplitude as mean voltage between 200–500 ms after stimulus presentation at 6 centro-parietal electrodes (Cz, C3, C4, Pz, P3, and P4), baseline-corrected by subtracting the mean amplitude in the −100–0 ms time window. The details of this dataset are described in Table 1. In this study, stimuli had either expected (highest-cloze) or unexpected (low-cloze) critical words.

#### Calculating the Metrics.

To investigate how well the language models’ predictions correlate with N400 amplitude, we ran each of the stimulus sentences from the six studies (Federmeier et al., 2007; Hubbard et al., 2019; Nieuwland et al., 2018; Szewczyk & Federmeier, 2022; Szewczyk et al., 2022; Wlotko & Federmeier, 2012) up until the critical noun through each of the 37 language models, calculating surprisal for each of the critical words. As is commonly the case with transformer language models, not all critical nouns were in each model’s vocabulary as individual tokens. Because all models are autoregressive, calculating the surprisal of multi-token words is straightforward—we calculate the surprisal of each sub-word token of the critical word given the preceding context (including preceding sub-word tokens) and take their sum, which is equivalent to taking the product of the probabilities. These surprisal values were then exponentially transformed as previously described.

#### Procedure for Statistical Analysis.

In order to test how well each form of exponentially-transformed surprisal calculated using each language model predicts N400 amplitude, we construct linear mixed-effects regression models using these variables as predictors and N400 amplitude as the dependent variable, comparing the Akaike’s Information Criterion (AIC; Akaike, 1973) of these regressions. AIC provides a measure of a regression’s fit to the data, with a lower AIC indicating a better fit. We run further analyses on these AIC values to compare how well each metric performs across models.

When analyzing the data from the Nieuwland et al. (2018) study, our statistical analysis approach aimed to match the original as much as possible. In these models, N400 amplitude was the dependent variable. The variable of interest for each language model (i.e., surprisal^*k*^) was included as a fixed effect. The original study was carried out at multiple laboratories, with previous work showing that depending on the subset of the data used, laboratory can be a significant predictor of N400 (Michaelov et al., 2022; Nieuwland et al., 2018), so we also included this as a fixed effect. In order to be able to compare regression fit across language models and metrics, the random effects structure needed to be consistent across regressions, and the maximal random effects structure that fulfiled this requirement in addition to converging and not resulting in any singular fits included a random intercept for each subject. All numerical variables were z-scored.

The 5 other datasets analyzed (Federmeier et al., 2007; Hubbard et al., 2019; Nieuwland et al., 2018; Szewczyk & Federmeier, 2022; Szewczyk et al., 2022; Wlotko & Federmeier, 2012) were all preprocessed in the same way by Szewczyk and Federmeier (2022). We kept our statistical analysis as close to those in Szewczyk and Federmeier (2022) as possible. As in Szewczyk and Federmeier (2022), un-baselined N400 amplitude was the dependent variable, with the baseline amplitude included as a fixed effect. The other covariates included in the original analyses and provided by Szewczyk and Federmeier (2022) were concreteness (Brysbaert et al., 2014), frequency (logarithmically transformed; Brysbaert & New, 2009), orthographic neighborhood (OLD20; Yarkoni et al., 2008), and sentence position, which we also included as fixed effects. We included the maximal random effects structure that would allow model convergence, result in no singular fits, and be consistent across regressions. The resulting random effects structure included random slopes for the baseline voltage for each subject and item, as well as a random intercept for each item. All numerical values were z-scored.

**Figure 1.**
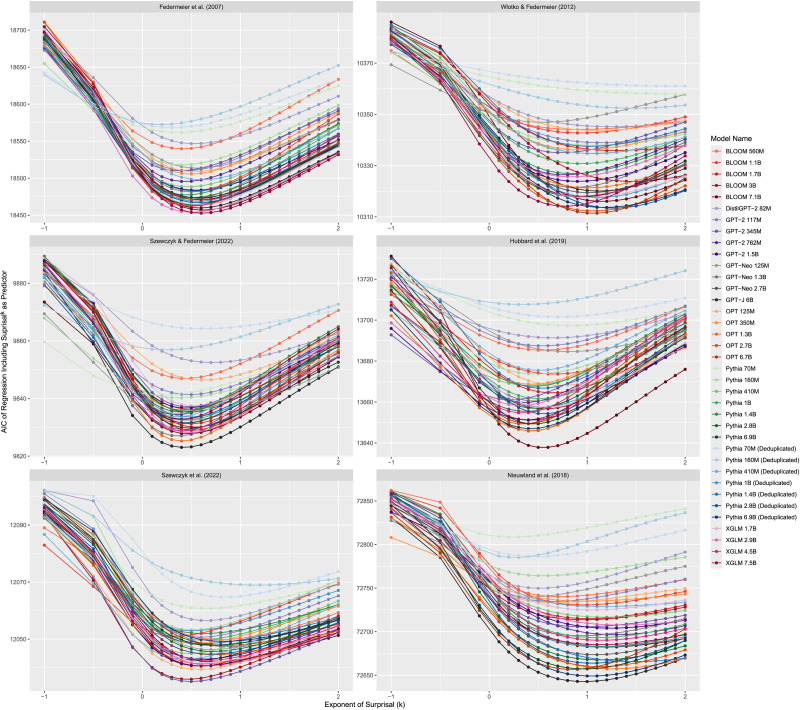
AIC of regressions predicting N400 amplitude with the exponentiated values of the surprisal calculated using 37 autoregressive transformer language models.

All graphs were created and statistical analyses carried out in *R* (R Core Team, 2023) using *Rstudio* (RStudio Team, 2020) and the *tidyverse* (Wickham et al., 2019), *lme4* (Bates et al., 2015), *lmerTest* (Kuznetsova et al., 2017), *mgcv* (Wood, 2017), *ggh4x* (van den Brand, 2021), *tidytext* (Silge & Robinson, 2016), *ggtext* (Wilke & Wiernik, 2022), *RColorBrewer* (Neuwirth, 2022), and *osfr* (Wolen et al., 2020) packages. All figures except Figure 1 use colorblind-friendly palettes (Chang, 2022). All reported *p*-values are corrected for multiple comparisons based on false discovery rate (Benjamini & Yekutieli, 2001) across all statistical tests carried out. We provide all data, code, and statistical analysis scripts at https://osf.io/w5hez.

### Results

The fit of all regressions including surprisal^*k*^ are shown in Figure 1. For four of the six datasets (Federmeier et al., 2007; Hubbard et al., 2019; Szewczyk & Federmeier, 2022; Szewczyk et al., 2022), the lowest AICs are achieved by regressions with sub-linear transformations of surprisal as the predictor of N400 amplitude, indicating that these best fit the human data. For the remaining two datasets (Nieuwland et al., 2018; Wlotko & Federmeier, 2012), the results are less clear—for the majority of language models, the best transformation of surprisal appears to be at *k* = 1 (i.e., no transformation) or slightly above.

To investigate these results further, we fit a general additive model to these AIC values for each dataset, predicting them using the default thin plate regression splines provided by the *mgcv* (Wood, 2017) package to fit *k* as a predictor and including random effects terms for each language model. All fitted GAMs had adjusted *R*^2^ values of greater than 0.9 (Federmeier et al., 2007: adjusted *R*^2^ = 0.94; Wlotko & Federmeier, 2012: adjusted *R*^2^ = 0.92; Szewczyk & Federmeier, 2022: adjusted *R*^2^ = 0.93; Hubbard et al., 2019: adjusted *R*^2^ = 0.90; Szewczyk et al., 2022: adjusted *R*^2^ = 0.96; Nieuwland et al., 2018: adjusted *R*^2^ = 0.92).

These general additive models were used to estimate the power of surprisal that produces the lowest AIC across language models, after accounting for differences between models. To do this, we generate a dummy dataset with values of *k* between 0 and 2 at 0.1 increments and an arbitrary (non-existent) language model. The general additive models were then used to estimate the regression AICs for an arbitrary language model based on *k* alone. The results replicate the numerical descriptions above—the general additive models estimate the lowest AIC for four of the datasets to occur when 0 < *k* < 1 (Federmeier et al., 2007: *k* = 0.5; Hubbard et al., 2019: *k* = 0.5; Szewczyk et al., 2022: *k* = 0.6; Szewczyk & Federmeier, 2022: *k* = 0.4). Also matching the graphs, the lowest AIC for the Nieuwland et al. (2018) and Wlotko and Federmeier (2012) datasets is estimated to occur when *k* = 1.

We also see a similar pattern if we look at the actual best-fitting predictability metric for each dataset overall: for the Federmeier et al. (2007) dataset this is surprisal^0.6^ as calculated by XGLM-7.5B, for Hubbard et al. (2019) this is BLOOM 7.1B surprisal^0.5^, for Szewczyk et al. (2022) this is GPT-2 345M surprisal^0.5^, for Szewczyk and Federmeier (2022) this is GPT-J 6B surprisal^0.4^, for Nieuwland et al. (2018) this is GPT-J 6B surprisal^1^ (i.e., surprisal), and for Wlotko and Federmeier (2012) this is OPT 6.7B surprisal^1.1^.

### Discussion

The results of the analysis are clear: for four of the six datasets (Federmeier et al., 2007; Hubbard et al., 2019; Szewczyk & Federmeier, 2022; Szewczyk et al., 2022), the value of *k* that leads to the surprisal^*k*^ that best fits N400 amplitude is well below 1—in fact, they are closer to 0.5. Thus, for these datasets, the results suggest that there is a sub-linear relationship between surprisal and N400 amplitude; that is, a sub-logarithmic relationship between probability and the N400. For the remaining 2 datasets, (Nieuwland et al., 2018; Wlotko & Federmeier, 2012), the best values of *k* are close to 1, suggesting a linear relationship between surprisal and the N400, and so a logarithmic relationship between probability and the N400.

These results depart from previous work on reading time, where, depending on the dataset and method of analysis, the best values of *k* tend to fall on both sides of 1 (Shain et al., 2024), or even tend to be greater than 1 (Hoover et al., 2023; Meister et al., 2021), supporting the logarithmic or super-logarithmic accounts respectively. By contrast, in the present study, the evidence leans in the opposite direction—while two datasets appear to support a logarithmic relationship, the remaining four support a sub-logarithmic relationship. As far as we are aware, this is the first study with evidence most strongly supporting a sub-logarithmic relationship between contextual probability and processing difficulty.

## ANALYSIS 2: A COMPARISON OF METRICS AND LANGUAGE MODELS

### Introduction

In Analysis 1, we sought to quantify which exponential transformation of surprisal best predicts the N400, testing whether a logarithmic, sub-logarithmic, or super-logarithmic relationship between probability and the N400 best explains the data. We found that for four of the six datasets (Federmeier et al., 2007; Hubbard et al., 2019; Szewczyk & Federmeier, 2022; Szewczyk et al., 2022), a sub-logarithmic transformation best predicts N400 amplitude, while the results for the other two datasets suggest a logarithmic relationship (Nieuwland et al., 2018; Wlotko & Federmeier, 2012). However, the approach in Analysis 1 raises two questions, which we address in this section.

First, how meaningful are the patterns observed in Analysis 1? Because the approach only estimates the overall best exponent from all language models, it is not clear how much better the best exponential transformation is compared to the alternatives, or how consistent any patterns are across language models. In this second analysis, we address both concerns, comparing surprisal with the best sub-logarithmic metric overall, which we find to be surprisal^0.6^.

The second question is whether the previous finding that language model surprisal is a better predictor of N400 amplitude than language model probability (Szewczyk & Federmeier, 2022; Yan & Jaeger, 2020) holds for the larger number of language models and datasets that we test. As previously discussed, two studies thus far have directly compared how probability and surprisal predict N400 amplitude, finding surprisal to be a better predictor (Szewczyk & Federmeier, 2022; Yan & Jaeger, 2020). However, as noted, these studies only use one language model each. In this analysis, in addition to comparing the fit of surprisal and surprisal^0.6^ to N400 amplitude, we also compare the fit of probability to both of these. Thus, we expand upon previous work by comparing how well probability and surprisal predict N400 amplitude by analyzing the predictions of 37 contemporary transformer language models, as well as carrying out the first analysis comparing the performance of either of these metrics to sub-logarithmically transformed probability (i.e., surprisal^0.6^).

### Method

Our analyses used the same datasets and language models as in Analysis 1. To calculate the best sub-logarithmic metric overall, we return to general additive models as in Analysis 1, but instead fit a single model to predict regression AIC across all datasets (including dataset as a random effect). When we construct a dummy dataset as in Analysis 1, we find that the additive model predicts the best (lowest) AIC overall to occur with surprisal^0.6^, so we use this as our best sub-logarithmic metric. We then calculate probability, surprisal, and surprisal^0.6^ for each stimulus in each dataset using each language model.

### Results

**Figure 2.**
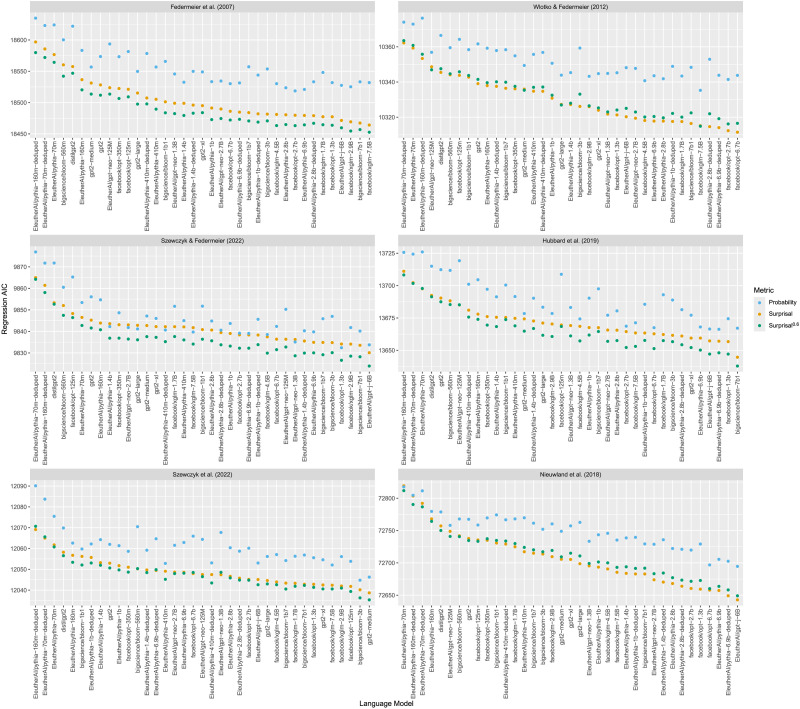
AIC of regressions predicting N400 amplitude using the probability, surprisal, or surprisal^0.6^ calculated by 37 autoregressive transformer language models.

The AICs of the regressions including probability, surprisal, or surprisal^0.6^ are presented in Figure 2. As can be seen visually, on the whole, the regressions including surprisal as a predictor perform better than those including probability. In addition, we see that regressions including surprisal^0.6^ perform better than those including probability. As in Analysis 1, we see that for four out of six datasets (Federmeier et al., 2007; Hubbard et al., 2019; Szewczyk & Federmeier, 2022; Szewczyk et al., 2022), surprisal^0.6^ is a better predictor of N400 amplitude than surprisal, while the reverse is true for the remaining two datasets (Nieuwland et al., 2018; Wlotko & Federmeier, 2012).

We next quantify the exact degree to which the AICs of regressions including probability, surprisal, and surprisal^0.6^ differ. To do this, we constructed linear mixed effects models comparing the performance of each pair of metrics (probability and surprisal, probability and surprisal^0.6^, and surprisal and surprisal^0.6^) as predictors. These linear mixed-effects models all had regression AIC as the dependent variable, metric as the predictor, and language model as a random intercept. We show the linear mixed effects models’ estimates of the difference in AIC between regressions with each metric as a predictor in Table 2. A difference in AIC of 4 or more is generally taken to indicate a ‘substantial’ difference in support of the regression with the lower AIC over that with the higher AIC (Burnham & Anderson, 2004). Thus, we see that for all datasets, regressions using surprisal or surprisal^0.6^ as a predictor tend to fit the N400 data substantially better than those using probability as a predictor. When comparing surprisal and surprisal^0.6^, however, the results are less clear—for three of the datasets (Federmeier et al., 2007; Hubbard et al., 2019; Szewczyk & Federmeier, 2022), surprisal^0.6^ as a predictor tends to lead to regressions that better fit the N400 data than surprisal, while for the Nieuwland et al. (2018) dataset, the reverse is true. The estimated differences in AICs between regressions including surprisal and surprisal^0.6^ as predictors on the remaining two N400 datasets (Szewczyk et al., 2022; Wlotko & Federmeier, 2012) are less than 4, and thus it is not clear that there is a meaningful difference between the two metrics on these datasets.

**Table 2.** Estimated differences between the AICs of regressions using probability, surprisal, and surprisal^0.6^ as predictors. **P-S** reflects the difference between the AICs of regressions with probability (*P*) and surprisal (*S*) as predictors where the value reflects the extent to which regressions with surprisal as a predictor have a lower AIC than regressions with probability as a predictor. In the same way, **P-S**^0.6^ reflects the difference in AIC between probability (*P*) and surprisal^0.6^ (*S*^0.6^) and **S-S**^0.6^ the difference in AIC between surprisal (*S*) and surprisal^0.6^ (*S*^0.6^).

**Dataset**	**P-S**	**P-S^0.6^**	**S-S^0.6^**
Nieuwland et al. (2018)	41.91	37.53	−4.38
Federmeier et al. (2007)	51.85	66.15	14.30
Wlotko and Federmeier (2012)	22.44	20.05	−2.39
Szewczyk and Federmeier (2022)	5.72	10.86	5.14
Hubbard et al. (2019)	18.23	24.43	6.19
Szewczyk et al. (2022)	11.89	13.41	1.52

In order to test how robust these estimates are, we carried out pairwise supplementary analyses evaluating whether metric (i.e., probability vs. surprisal, probability vs. surprisal^0.6^, or surprisal vs. surprisal^0.6^) was a significant predictor of regression AIC by running likelihood ratio tests comparing the aforementioned linear mixed-effects models to equivalent models not including metric as a predictor. The results are reported in Appendix A. In all cases, metric was a significant predictor, and thus the findings that using surprisal^0.6^ to predict N400 amplitude leads to the regression with the best fit on 3 of the datasets (Federmeier et al., 2007; Hubbard et al., 2019; Szewczyk & Federmeier, 2022) and that using surprisal leads to the regressions with the best fit on the Nieuwland et al. (2018) dataset are statistically significant.

### Discussion

This analysis has three findings. First, we find that the results of Szewczyk and Federmeier (2022) and Yan and Jaeger (2020) generalize across a larger number of models—overall, surprisal is a better predictor of N400 amplitude than probability is. Second, we find that, across language models, surprisal^0.6^ is also a better predictor of N400 amplitude than probability is. Finally, when we compare the performance of regressions predicting N400 amplitude using surprisal or surprisal^0.6^, we provide additional support for the results of Analysis 1: for the majority of datasets (Federmeier et al., 2007; Hubbard et al., 2019; Szewczyk & Federmeier, 2022; Szewczyk et al., 2022), using surprisal^0.6^ to predict N400 amplitude leads to a numerically lower AIC than using surprisal; while for the remaining datasets (Nieuwland et al., 2018; Wlotko & Federmeier, 2012), the reverse is true.

Analyzing the differences in more detail and running statistical tests adds further nuance to these results. Specifically, on three of the datasets (Federmeier et al., 2007; Hubbard et al., 2019; Szewczyk & Federmeier, 2022), using surprisal^0.6^ to predict N400 amplitude tends to lead to a substantially better fit, and this difference is statistically significant across language models. In addition, for one dataset (Nieuwland et al., 2018), the substantially better fit to the data of surprisal (compared to surprisal^0.6^) is statistically significant. Finally, for the remaining two datasets, while there are significant differences, the effect size of under 4 AIC suggests that there is no clear difference in whether using surprisal or surprisal^0.6^ to predict N400 amplitude leads to a better fit to the data.

## ANALYSIS 3: VARIANCE EXPLAINED

### Introduction

A striking result of the present study so far has been that in contrast to work on reading time, the evidence best supports a sub-logarithmic relationship between probability and N400 amplitude. However, thus far, we have only analyzed differences in overall fit between models; the key quantitative question is to what extent the variables discussed can explain variance in N400 amplitude. That is the question we address in this section.

To do this, we turn to our best-performing language models. If we hope to understand the mathematical relationship between contextual probability and the N400, we should use the models whose metrics (i.e., transformed probability values) most closely correlate with N400 amplitude to avoid confounds. For example, because surprisal magnifies differences at the low end of the scale (i.e., when probability is close to zero), surprisal may magnify small differences in predictions across language models such that probabilities from poorly performing language models might actually spuriously outperform surprisal in some cases.

We therefore select the best overall language model for each dataset, and test how well probability, surprisal, and surprisal^0.6^ each explain the variance in N400 amplitude in that dataset.

### Method

Based on the results of Analyses 1 and 2, we use surprisal^0.6^ as our sub-logarithmic metric. We select the best language models by looking at those that produced the regression with the lowest AIC for each dataset in Analysis 1: these were XGLM 7.5B on the Federmeier et al. (2007) dataset, BLOOM 7.1B on Hubbard et al. (2019), GPT-2 345M on Szewczyk et al. (2022), OPT 6.7B on Wlotko and Federmeier (2012), and GPT-J 6B on Nieuwland et al. (2018) and Szewczyk and Federmeier (2022).

### Results

**Figure 3.**
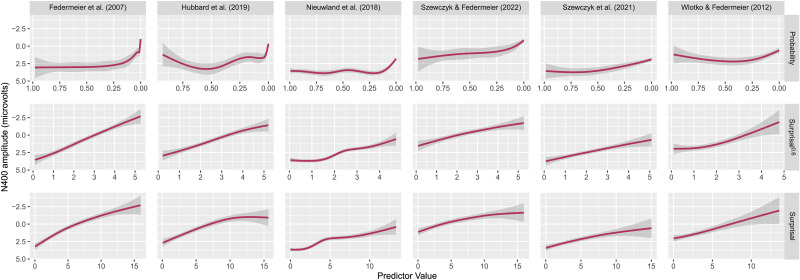
N400 amplitude as a function of GPT-J 6B probability, surprisal, and surprisal^0.6^. The x-axis for probability is reversed for easier comparison with surprisal and surprisal^0.6^.

First, we visualize the relationship between each metric and the N400. Figure 3 shows the relationship between baselined N400 amplitude and GPT-J 6B probability, surprisal, and surprisal^0.6^ for all datasets. In line with the results of Analysis 2, we see that for the majority of datasets (Federmeier et al., 2007; Hubbard et al., 2019; Szewczyk & Federmeier, 2022; Szewczyk et al., 2022), surprisal^0.6^ does indeed appear to have the most (approximately) linear relationship to N400 amplitude. By contrast, surprisal appears to have the most linear relationship with N400 amplitude in the Wlotko and Federmeier (2012) dataset. Finally, the results for the Nieuwland et al. (2018) are less clear—visually, it is hard to tell which metric has the most linear relationship to N400 amplitude. We provide the equivalent graphs for the other language models in Appendix B.

**Figure 4.**
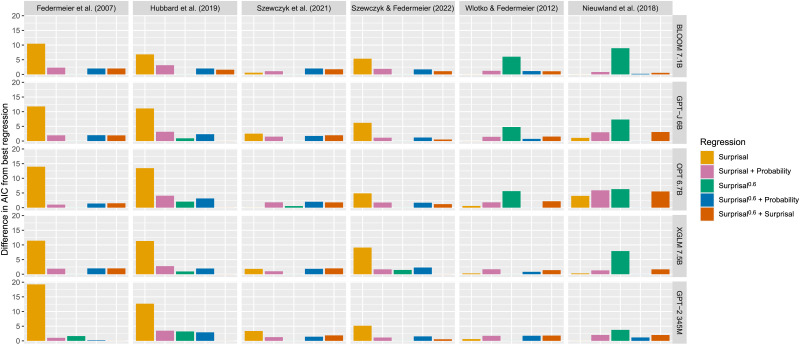
The fit of regressions including probability, surprisal, surprisal^0.6^, probability and surprisal, probability and surprisal^0.6^, and surprisal and surprisal^0.6^ as predictors of N400 amplitude. We look at the results for the 5 language models that best predict each of the 6 datasets.

We next investigate the extent to which each metric explains variance in N400 amplitude, and whether any variables explain additional variance once others are accounted for. Thus, in addition to looking at probability, surprisal, and surprisal^0.6^ as in Analysis 2, we also look at the combination of surprisal and probability, following Szewczyk and Federmeier (2022). We show how the fit of regressions including these predictors compare in Figure 4. Given the drastically worse performance of the probability-only regressions on some of the datasets (in particular Federmeier et al., 2007; Nieuwland et al., 2018), we exclude these from the Figure 4; however, for completeness, we provide these in Appendix C.

We then test how well each of these predictors or sets of predictors calculated using each language model explains variance in N400 amplitude using likelihood ratio tests between linear mixed-effects models, testing the effect of adding probability, surprisal, surprisal^0.6^, and both probability and surprisal to a regression already including one of these, thereby testing whether the added variable explains additional variance. Specifically, we test the effect of adding surprisal^0.6^ to a linear mixed-effects model already including surprisal (*S* + *S*^0.6^), adding probability to a model already including surprisal (*S* + *P*), adding surprisal to a model already including surprisal^0.6^ (*S*^0.6^ + *S*), adding probability to a model already including surprisal^0.6^ (*S*^0.6^ + *P*), adding surprisal to a model already including probability (*P* + *S*), adding surprisal^0.6^ to a model already including probability (*P* + *S*^0.6^), adding surprisal^0.6^ to a model already including surprisal and probability ((*S* + *P*) + *S*^0.6^), and adding both surprisal and probability to a model already including surprisal^0.6^ (*S*^0.6^ + (*S* + *P*)).

The results for the predictors calculated using GPT-J 6B are presented in Table 3. As previously noted, all *p*-values were corrected for multiple comparisons using the stringent false discovery rate method proposed by Benjamini and Yekutieli (2001), ensuring that we report only the most robust effects. The tables for the other 5 models are provided in Appendix D.

**Table 3.** Results of the likelihood ratio tests testing the effect of adding GPT-J 6B probability (*P*), surprisal (*S*), surprisal^0.6^ (*S*^0.6^), or a combination of these to a linear mixed-effects model already including one or more other of these variables, thereby testing whether they explain any additional variance. As an example, *S* + *P* refers to a likelihood ratio test of whether probability explains additional variance in N400 amplitude above and beyond that explained by surprisal. FWOK07 refers to Federmeier et al. (2007), WF12 to Wlotko and Federmeier (2012), SF22 to Szewczyk and Federmeier (2022), HRJF19 to Hubbard et al. (2019), SMF22 to Szewczyk et al. (2022), and N18 to Nieuwland et al. (2018).

**Dataset**	***S*^0.6^ + *S***		***S*^0.6^ + *P***		***S* + *S*^0.6^**		***S* + *P***	
	***χ*^2^(1)**	***p***	***χ*^2^(1)**	***p***	***χ*^2^(1)**	***p***	***χ*^2^(1)**	***p***
FWOK07	0.08	1.0000	0.03	1.0000	**11.87**	**0.0116**	**11.85**	**0.0116**
WF12	5.25	0.2676	6.05	0.1827	0.49	1.0000	0.59	1.0000
SF22	1.50	1.0000	0.82	1.0000	7.69	0.0813	7.06	0.1104
HRJF19	2.91	0.9155	0.59	1.0000	**13.1**	**0.0064**	**9.95**	**0.0254**
SMF22	0.05	1.0000	0.27	1.0000	2.55	1.0000	3.03	0.867
N18	6.26	0.1652	**9.31**	**0.0349**	0.01	1.0000	0.08	1.0000
**Dataset**	***P* + *S***		***P* + *S*^0.6^**		**(*S* + *P*) + *S*^0.6^**		***S*^0.6^ + (*S* + *P*)**	
	***χ*^2^(1)**	***p***	***χ*^2^(1)**	***p***	***χ*^2^(1)**	***p***	***χ*^2^(2)**	***p***
FWOK07	**67.88**	**<0.0001**	**67.85**	**<0.0001**	0.13	1.0000	0.20	1.0000
WF12	**28.46**	**<0.0001**	**29.16**	**<0.0001**	0.14	1.0000	5.49	0.6927
SF22	**10.73**	**0.0182**	**10.67**	**0.0184**	0.71	1.0000	1.58	1.0000
HRJF19	**19.11**	**0.0003**	**19.94**	**0.0002**	6.19	0.1697	5.95	0.5716
SMF22	**10.96**	**0.0164**	**10.71**	**0.0182**	0.47	1.0000	1.00	1.0000
N18	**51.87**	**<0.0001**	**54.85**	**<0.0001**	1.76	1.0000	8.09	0.2217

**Figure C1.**
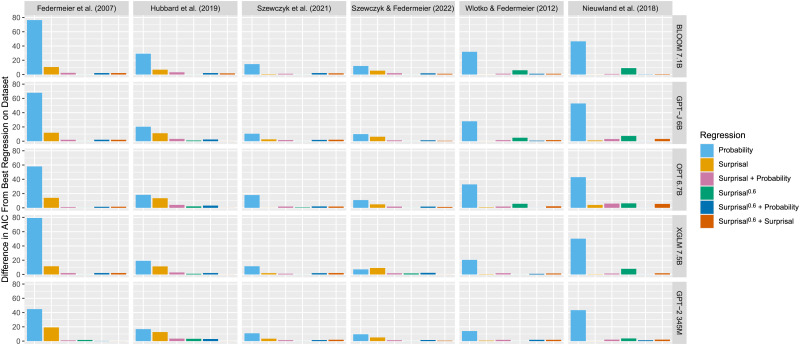
The fit of regressions including probability, surprisal, surprisal^0.6^, and combinations of these as predictors of N400 amplitude. We look at the results for the 5 language models that best predict each of the 6 datasets.

First, and perhaps least surprising, is the finding that in all the datasets both surprisal and surprisal^0.6^ explain a significant amount of variance in N400 amplitude above and beyond that explained by probability. This is in line both with previous work and the results presented in Figure C1, where we see that probability is a substantially worse predictor on its own than the other two metrics. As can be seen in Figure C1, this is also the case with the other 4 language models. However, there is one exception to this pattern—the difference for XGLM 7.5B on the Szewczyk and Federmeier (2022) dataset is not significant.

Next, we see that probability explains variance in N400 amplitude above and beyond that explained by surprisal^0.6^ on the Nieuwland et al. (2018) dataset. This is also the case with probabilities calculated using BLOOM 7.1B and XGLM 7.5B, but not OPT 6.7B or GPT-2 345M. It is also worth noting that on the same dataset, BLOOM 7.1B surprisal explains variance in N400 amplitude above and beyond that explained by surprisal^0.6^. As can be seen in Figure 4, in each of these cases, the combined surprisal^0.6^ and probability regression has a lower AIC than the equivalent combined surprisal and probability regression, with the difference in AIC exceeding 4 in the case of OPT 6.7B.

We also see that both probability and surprisal^0.6^ explain variance above and beyond that explained by surprisal on the Federmeier et al. (2007) and Hubbard et al. (2019) datasets. This is also true for other models with the exception of BLOOM 7.1B probabilities, with which only the Federmeier et al. (2007) dataset shows the effect. In addition, the effect is also found on the Szewczyk and Federmeier (2022) dataset for XGLM 7.5B probabilities.

Finally, while we see differences between how well they predict N400 amplitude numerically in Figure 4, after correction for multiple comparisons, we do not find surprisal^0.6^ to explain any variance not already explained by both surprisal and probability, and neither do we find the reverse. This suggests that surprisal^0.6^ and the combination of surprisal and probability explain very similar variance in N400 amplitude.

### Discussion

Several clear results arise from these analyses. First, surprisal predicts N400 amplitude better than probability does. For five language models—each of which is the model that best predicts N400 amplitude on at least one dataset—surprisal explains a significant amount of the variance in N400 amplitude not explained by probability in every dataset, even after correction for multiple comparisons. Thus, these results replicate and expand on those of Szewczyk and Federmeier (2022). Specifically, Szewczyk and Federmeier (2022) find that GPT-2 1.5B surprisal explains variance in N400 amplitude not explained by probability on a combined dataset made up of four datasets (Hubbard et al., 2019; Szewczyk & Federmeier, 2022; Szewczyk et al., 2022; Wlotko & Federmeier, 2012), as well as for the unexpected (cloze ≤ 5%) completions in the Federmeier et al. (2007) dataset. We find the same pattern to hold when all the data from each of these datasets (as well as Federmeier et al., 2007 and Nieuwland et al., 2018) are analyzed separately using the probabilities calculated from five additional language models (GPT-J 6B, BLOOM 7.1B, OPT 6.7B, XGLM 7.5B, and GPT-2 345M). Thus, our results suggest that this result is generalizable across language models and on at least one entirely new dataset (Nieuwland et al., 2018).

We also find that for two of the datasets (Federmeier et al., 2007; Hubbard et al., 2019), probability conversely explains variance in N400 amplitude not explained by surprisal. Szewczyk and Federmeier (2022) report this for their aforementioned combined dataset, as well as the expected completions from Federmeier et al. (2007). Our results expand upon the latter finding. This effect is present with the probabilities calculated using five additional language models, even when considering all data from Federmeier et al. (2007)—i.e., not just expected completions—and even when correcting for multiple comparisons. Our results also pinpoint the Hubbard et al. (2019) dataset as a likely source of the effect on the combined dataset—we see that the effect of probability significantly predicts N400 amplitude even when surprisal is accounted for with four of the five models.

These results raise the question of why we only see this pattern for two of the six datasets. One possibility is the experimental stimuli themselves—of the five datasets for which the stimulus selection process is reported, only Hubbard et al. (2019) directly use stimuli from Federmeier et al. (2007) without adding additional stimili; and thus there may be something about these stimuli that leads to the effect being detected. Whether this is because the effect is present but undetectable in the other stimuli or is caused by some as yet unidentified feature of the Federmeier et al. (2007) stimuli is a question for further research. However, it is worth noting that one piece of evidence in favor of the former is that one of the datasets that does not show this effect is the Nieuwland et al. (2018), which has the smallest number of items and is predominantly made up of high-constraint sentences only, and the other is Wlotko and Federmeier (2012), which has the smallest number of experimental participants.

We also find two novel results based around surprisal^0.6^. First, we find that like surprisal, surprisal^0.6^ explains a significant amount of the variance in N400 amplitude not explained by probability across virtually all language models and datasets. In addition, we find that for the datasets where probability explains the variance in N400 amplitude above and beyond that explained by surprisal, surprisal^0.6^ also explains variance not explained by surprisal; and in addition, probability does not explain variance not explained by surprisal^0.6^. In fact, with the exception of the Nieuwland et al. (2018) dataset, surprisal^0.6^ explains all the statistically significant variance explained by both surprisal and probability across models and datasets. It is still important to note, however, that with the Nieuwland et al. (2018) dataset, GPT-J 6B, BLOOM 7.1B, and XGLM 7.5B (but not OPT 6.7B or GPT-2 345M) probability explains variance in N400 amplitude not explained by surprisal^0.6^, as does BLOOM 7.1B surprisal. Given the aforementioned limitations of the Nieuwland et al. (2018) dataset and the fact that this is only the case for three of the five language models on one of the six datasets tested, it is possible that this is simply an anomalous result, but this is something that would need to be tested by running similar analyses on a larger number of additional datasets. Taken together, our results for surprisal^0.6^ may be taken to suggest that the individual effects of probability and surprisal reported by Szewczyk and Federmeier (2022) and replicated in our work could instead be empirically accounted for by a single sub-logarithmic relationship (i.e., (−*log*(*p*)))^0.6^ between language model probability and N400 amplitude. However, we are not aware of any previous theoretical work predicting such a relationship.

Overall, the results of this analysis showed three things. First, we replicate and expand Szewczyk and Federmeier’s (2022) finding that language model surprisal explains variance in N400 amplitude above and beyond that explained by probability. We also replicate and extend the finding that probability explains variance not explained by surprisal on the Federmeier et al. (2007) and Hubbard et al. (2019) datasets. Finally, we found both of these effects are generally captured by a single variable—surprisal^0.6^. In all cases, all the variance explained by surprisal that is not explained by probability is explained by surprisal^0.6^, and all variance in Federmeier et al. (2007) and Hubbard et al. (2019) explained by probability that is not explained by surprisal is explained by surprisal^0.6^. In fact, we see that on 27 of the 30 combinations of language models and datasets, surprisal^0.6^ explains all the variance in N400 amplitude explained by either surprisal, probability, or their combination. Finally, in the 3 remaining cases, the combination of surprisal^0.6^ and probability predicts N400 amplitude at least as well as the combination of surprisal and probability.

## INTERIM DISCUSSION

The results of Analyses 1–3 show that the best single predictor of N400 amplitude is sub-logarithmically transformed probability, and that the same variance is explained by a combination of probability and surprisal. Thus, the empirical results can be considered to equally support a sub-logarithmic relationship or the combined relationship (in line with the multiple sub-component account of Szewczyk & Federmeier, 2022). Given the lack of any previously-proposed theory accounting for the sublogarithmic relationship, in this paper we focus on this new result and how it compares to Szewczyk and Federmeier’s (2022) multiple sub-component account. Thus, we do not consider the additional possibilities of multiple sub-component accounts involving a sub-logarithmic relationship as well as either surprisal, probability, or both.

The present study shows the difficulty in distinguishing empirically between the sublogarithmic and multiple sub-component relationships—both predict N400 amplitude well. Both also explain seemingly disparate findings in previous work. For example, one well-established phenomenon is that N400 amplitude can differ greatly between words with matched cloze probabilities, especially between low (or zero) cloze items (DeLong et al., 2019; Federmeier & Kutas, 1999; Metusalem et al., 2012). More recent work, however, suggests that at least some of this variance in N400 amplitude can be captured by language model surprisal (Michaelov & Bergen, 2020, 2022). Surprisal’s success with low-probability words likely derives from the fact that it emphasizes differences in probability at the low end of the scale. But by the same token, it also reduces the differences at the high end of the probability scale. Empirical results suggests that this could be a problem for modeling the N400—indeed, part of the empirical motivation for Szewczyk and Federmeier’s (2022) multiple sub-component account is that language model probability better predicts the amplitude of the N400 response elicited by high-probability items than language model surprisal does. The question, then, is how to determine whether the theoretically unexplained but parsimonious sublogarithmic relationship or the theoretically-motivated but more complex multiple sub-component account is more strongly supported by the evidence. This is the aim of the remainder of this paper.

### Towards a Multiple Sub-component Account

First, we consider the evidence that exists or would be need to exist to support the multiple sub-component account as proposed by Szewczyk and Federmeier (2022). This account has three aspects: the idea that the N400 is sensitive to both contextual predictability and similarity (or more specifically, overlap of semantic features in long-term memory); the empirical result that the N400 can be predicted by contextual probability and surprisal; and the linking hypothesis that the effect of contextual predictability on N400 amplitude can be operationalized by a linear relationship between contextual probability and N400 amplitude and that the effect of contextual similarity on N400 amplitude can be operationalized by a logarithmic relationship between contextual probability and N400 amplitude (i.e., a linear relationship between surprisal and N400 amplitude).

There is some evidence for the first of these. Lau et al. (2013), for example, investigate this question through experimental manipulation. Their study used the well-established word-pair priming paradigm under which words preceded by related words elicit smaller N400 responses than words preceded by unrelated words (see, e.g., Bentin et al., 1985; Holcomb, 1988; Kutas, 1993; Kutas & Hillyard, 1989; Rugg, 1985). Lau et al. (2013) found that participants who had been presented with a larger proportion of sentences where the words were related showed increased reductions in the N400 for associated targets, as well as a difference in onset latency and topographic distribution of the priming effect compared to participants who were presented with fewer related word pairs. Lau et al. (2013) follow previous work (Becker, 1980; Neely, 1977) in arguing that the greater degree of predictive validity in the high relatedness proportion stimuli is indicative of increased predictive processing, and thus, that the differences in the N400 effects between relatedness proportions are due to an effect of a predictive process. As noted, the differences based on this manipulation are dissociable in latency and topographic distribution from the baseline N400 effects in word-pair priming that are generally thought to arise from associative processes, and thus, the effects of prediction and association are argued to be distinct and hypothesized to possibly arise from ‘qualitatively different’ neurocognitive processes (Lau et al., 2013).

An alternative approach is to test how much variance is explained either by contextual predictability or semantic feature overlap. In studies of this type (Frank & Willems, 2017; Parviz et al., 2011), in addition to using the predictions of language models (or augmented language models) to operationalize predictability, researchers use the contextual similarity of word vectors to model semantic feature overlap to model the N400. Specifically, Parviz et al. (2011) used latent semantic analysis (LSA; Dumais et al., 1988; Landauer et al., 1998) to calculate word vectors representing the semantics of all the words in the experimental stimuli, operationalizing semantic feature overlap as the cosine distance between the word vector representing the critical word and a context vector made up of the elementwise product of all word vectors in the context. Frank and Willems (2017), on the other hand, use their own implementation of *word2vec* (Mikolov, Chen, et al., 2013; Mikolov, Sutskever, et al., 2013) to calculate word vectors, basing their metric of ‘semantic distance’ on the cosine similarity of the critical word and the sum of the vectors of the words in its context. In both cases, semantic feature overlap was found to predict variance in N400 amplitude above and beyond predictability, supporting the idea that the two may arise from distinct sub-processes. It is also worth noting that there are a number of other studies that have been argued to directly or indirectly support this perspective (see Federmeier, 2021 for review).

The second aspect of the multiple sub-component account presented by Szewczyk and Federmeier (2022) is an empirical one—the finding that probability and surprisal can explain separate variance in N400 amplitude. Szewczyk and Federmeier (2022) provide evidence of this for GPT-2 1.5B (XL) probability, and we provide evidence for this for BLOOM 7.1B, GPT-2 345M (Medium), GPT-J 6B, OPT 6.7B, and XGLM 7.5B.

The third and final aspect of the account—the linking hypothesis—is less well-evidenced, however. Intuitively, given the fact that both language model predictions and aggregated cloze responses can be formulated as probabilities, it seems natural to stipulate that they could be linearly related. And while there does not appear to be a large difference in the extent to which un-transformed or log-transformed cloze probabilities predict N400 amplitude (Aurnhammer et al., 2021; Michaelov et al., 2022; Szewczyk & Federmeier, 2022), the evidence appears to support a linear relationship between cloze probability and reading time (Brothers & Kuperberg, 2021), leading Brothers and Kuperberg (2021) to argue that cloze probabilities closely reflect the extent to which words are predicted in the brain (see also Smith & Levy, 2011) and that processing difficulty is linearly related to this degree of proportional (predictive) preactivation. The account presented by Szewczyk and Federmeier (2022) follows this intuition in that it argues that the extent to which words are predicted is linearly related to N400 amplitude. However, while Szewczyk and Federmeier (2022) quantify the correlation between cloze probability and GPT-2 1.5B probability for expected (cloze probability > 0.05) words (*r* = 0.72), this is not compared to the degree of correlation between GPT-2 1.5B surprisal and cloze probability in the same range. Thus, in principle, even if we fully accept the proportional preactivation account of Brothers and Kuperberg (2021), it is not a given that language model probability is more related to these ‘subjective probabilities’ (Smith & Levy, 2011) that have been argued to be linearly related to processing difficulty (Brothers & Kuperberg, 2021) than is language model surprisal, for example. This is an empirical question that can be directly tested, and we do, in Analysis 4.

There is also limited evidence for the second part of the linking hypothesis, namely, that language model surprisal can operationalize the degree of semantic featural overlap between a critical word and its context. The main issue is that the composite processing difficulty account provided by Szewczyk and Federmeier (2022) could in principle apply to any featural overlap between a word and its context. Indeed, the explanation is the same as that given in the original version of the account presented by Smith and Levy (2013) but with word fragments replaced by semantic features. Thus, while it is in principle a plausible account, there is no direct evidence that contextual similarity is well-correlated with language model surprisal, even if the latter does in fact in part reflect the former. One possible indirect piece of evidence for this component is found in Michaelov et al. (2024), where the semantic featural overlap between a word and its context was operationalized as the cosine similarity between the GloVe (Pennington et al., 2014) or fastText (Mikolov et al., 2018) word embedding for the critical word and the mean of the embeddings of the words in the context. Crucially, GPT-3 surprisal was found to fully account for the variance in N400 amplitude explained by either metric of semantic featural overlap, which is consistent with the idea that the two are strongly related, as proposed by Szewczyk and Federmeier (2022). However, it is worth noting that it is also the case that GPT-3 surprisal explains all the variance explained by cloze probability (Michaelov et al., 2024); and thus, the evidence for the linking hypthosis is far from conclusive. We thus also investigate this relationship in Analysis 4.

### Towards a Sublogarithmic Account

As previously noted, no current theoretical account predicts a sublogarithmic relationship between contextual probability and any metric of processing difficulty, including the N400. Despite this, not all the accounts are inconsistent with such a relationship between statistical probability (as operationalized by language model probability) and the N400. This becomes clear if we consider the two accounts that explicitly posit that words are preactivated in the brain to an extent that correlates with contextual probability, namely, the contextual probability (Brothers & Kuperberg, 2021) and distribution update (Aurnhammer & Frank, 2019b) accounts. The linear (in the case of the contextual probability account) and logarithmic (in the case of the distribution update account) relationships described by these accounts are posited to be a result of these relative degrees of preactivation, which can be mathematically described as a probability distribution over candidate stimuli—the distribution of ‘subjective probabilities’ (Smith & Levy, 2011). Crucially, then, while the aforementioned three accounts make claims about the relationship between subjective probabilities and processing difficulty, they do not make any claim about the relationship between statistical probabilities and subjective probabilities.

Statistical probability does not directly correspond to cloze probability (for discussion, see Brothers & Kuperberg, 2021; Michaelov et al., 2022; Smith & Levy, 2011), and so it is possible that the subjective probability of a word in context is represented in the brain such that there is a nonlinear relationship between it and the word’s statistical probability. In fact, such a relationship may be indirectly supported by previous work. Several researchers have argued that the same linguistic representations that underlie the predictions that occur during language comprehension are likely to be those drawn on when responding to the cloze task (Brothers & Kuperberg, 2021; Smith & Levy, 2011). It is therefore perhaps unsurprising that Brothers and Kuperberg (2021) find that the relationship between cloze and processing difficulty is best described as linear. Meanwhile, however, recent empirical work has established the relationship between language probability and behavioral metrics of processing difficulty such as reading time to be decidedly non-linear (Hoover et al., 2023; Meister et al., 2021; Shain et al., 2024; Smith & Levy, 2013; Wilcox et al., 2023).

Thus, it is perfectly possible that cloze probability and language model probability may have different relationships with N400 amplitude, and that the relationship between statistical probability (as approximated by language mode probability) and subjective word probability (as approximated by cloze probability) may be nonlinear. We test the viability of this possibility by investigating the relationship between language model probability and cloze probability.

The first thing to consider is that, as noted above, while behavioral evidence suggests a linear relationship between subjective probability and reading time (see Brothers & Kuperberg, 2021), the precise relationship between subjective probability and N400 amplitude is less clear. In Michaelov et al. (2022), cloze surprisal (i.e., log-transformed cloze probability) is found to predict N400 amplitude slightly better than un-transformed cloze probability in sentences where cloze > 0. Szewczyk and Federmeier (2022), on the other hand, report a slightly better performance from cloze probability for stimuli where cloze > 0.05.

For this reason, in the present study, we consider both possibilities—that the relationship between subjective probability and N400 amplitude is linear (as under the contextual probability account), and that the relationship is logarithmic (as under the distribution update account). Taken together, therefore, there are two possible ways in which the relationship between statistical probability and N400 amplitude is sublogarithmic.

The first of these is that the relationship between statistical probability and subjective probability is sublogarithmic, and the relationship between subjective probability and N400 amplitude is linear, as under the contextual probability account. This would explain the evidence we find for a sublogarithmic relationship between language model probability and N400 amplitude. Given that this possibility involves a linear relationship between subjective probability and N400 amplitude and our finding that the best single characterization of the relationship between language model probability and N400 amplitude is linearly related to (−log *p*)^0.6^ (i.e., surprisal^0.6^), we test how closely language model surprisal^0.6^ correlates with cloze probability compared to un-transformed probability and surprisal.

Next we turn to the other possibility, namely, that in addition to a sublogarithmic relationship between statistical probability and N400 amplitude, there is also a logarithmic relationship between subjective probability and N400 amplitude, in line with the distribution update account. In this case, then, the relationship between language model probability and cloze probability would be linearly related to *e*^(−log *p*)^0.6^^ (i.e., language model *e*^surprisal^0.6^^). We again compare how closely this correlates with cloze probability.

In summary, while our empirical results suggest that there may be a sublogarithmic relationship between statistical probability and N400 amplitude, this is not something that has been suggested in any previous work. However, most accounts theorize based on a relationship between subjective probability and N400 amplitude, which, as we note, is not the same as statistical probability. If cloze probability shows a linear relationship to language model surprisal^0.6^ (in line with the contextual probability account) or language model *e*^surprisal^0.6^^ (in line with the distribution update account), this would provide independent empirical support for the sublogarithmic relationship between statistical probability and N400 amplitude. Future work would be needed to investigate why such a nonlinearity might characterize (or at least approximate) the relationship between statistical probability and subjective probability; but to the best of our knowledge, only one study (Smith & Levy, 2011) has thus far studied the relationship between the two, and consequently, substantial work is needed in this area regardless. We hope that the investigations in Analysis 4 can help to further this much-needed line of research.

## ANALYSIS 4: CORRELATIONS OF PREDICTORS

### Introduction

In this analysis, we ask whether it is possible to determine whether the multiple sub-component account of Szewczyk and Federmeier (2022) or the sublogarithmic account proposed in the Interim Discussion is better supported by the evidence. As noted in the Interim Discussion, under the multiple sub-component account of Szewczyk and Federmeier (2022), we should expect that un-transformed statistical probability (operationalized by language model probability) is linearly related to subjective probability (operationalized as cloze probability) and language model surprisal is logarithmically related to semantic featural overlap with the context (as operationalized based on similarity between word vectors). Meanwhile, if the sublogarithmic account is true and the sublogarithmic relationship approximates a component of the relationship between statistical probability and subjective probability, then the relationship between language model probability and subjective probability should be either sublogarithmic ((−log*p*)^0.6^; i.e., surprisal^0.6^) under the contextual probability account or an exponentation of this (*e*^(−log *p*)^0.6^^; i.e., *e*^surprisal^0.6^^) under the distribution update account. This is what we test in this section.

### Method

The N400 data and language models are the same as in Analysis 3. We also utilize the cloze probabilities provided by the authors (Nieuwland et al., 2018; Szewczyk & Federmeier, 2022) for each item.

In addition, we calculate the featural overlap between critical words and their preceding context. To do this, we follow Ettinger et al. (2016) and Michaelov et al. (2024), calculating the cosine similarity between the mean of the word embeddings of the context and the word embedding of the critical word. Based on their performance at predicting N400 amplitude in previous work (Michaelov et al., 2024), we use fastText (Joulin et al., 2017) word embeddings. Departing from Michaelov et al. (2024), we use the 300-dimensional embeddings trained on a combination of English Common Crawl and Wikipedia data from Grave et al. (2018) because the authors provide the original model, allowing the word embedding of any critical or context word to be calculated.

### Results

#### Correlation with Cloze.

**Figure 5.**
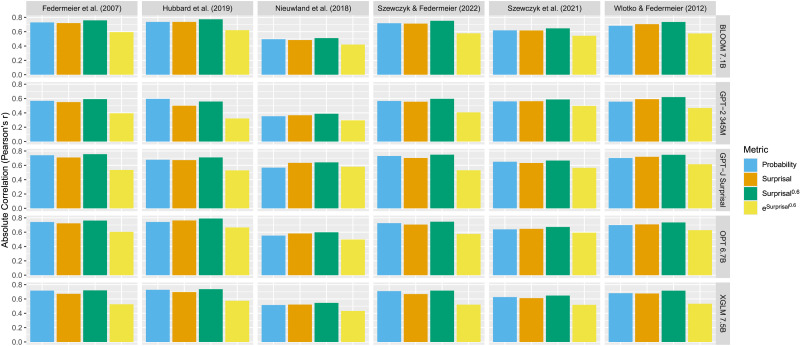
The absolute correlation coefficient between the probability, surprisal, surprisal^0.6^, and *e*^surprisal^0.6^^ calculated from each language model and cloze probability. This analysis only includes data from stimuli with a cloze probability greater than 0.05.

First, we test how closely cloze probability correlates with the probability, surprisal, surprisal^0.6^, and *e*^surprisal^0.6^^ calculated from each language model. To do this we calculate the absolute value of the correlation coefficient (Pearson’s *r*) of cloze probability and each of the aforementioned variables. Following Szewczyk and Federmeier (2022), we only look at words with a cloze probability of greater than 0.05. The results are shown in Figure 5.

These results align reasonably well with our formulation of Szewczyk and Federmeier’s (2022) multiple sub-component account. Under this account, we would expect probability to correlate more closely with cloze probability than surprisal does. This is indeed the case for a narrow majority of the comparisons—19 of the total 30. Intriguingly, the exceptions seem to align with those found in the previous analyses of this paper—8 of the 11 cases where surprisal is more closely correlated with cloze probability are the Nieuwland et al. (2018) and Wlotko and Federmeier (2012) datasets. Given that this analysis was carried out on the properties of the stimuli alone, this suggests that the fact that these datasets show different patterns in the N400 to the other datasets is likely due to the stimuli themselves.

We also tested how closely surprisal^0.6^ and *e*^surprisal^0.6^^ correlate with cloze probability. The results of this comparison are clearer—with the exception of GPT-J 6B surprisal on the Nieuwland et al. (2018) dataset, *e*^surprisal^0.6^^ is least closely correlated with cloze probability; and in all cases, surprisal^0.6^ is mostly closely correlated with cloze probability. Overall, then, the results of this analysis most strongly support the sub-logarithmic variant of the proportional preactivation account.

#### Correlation with Contextual Similarity.

**Figure 6.**
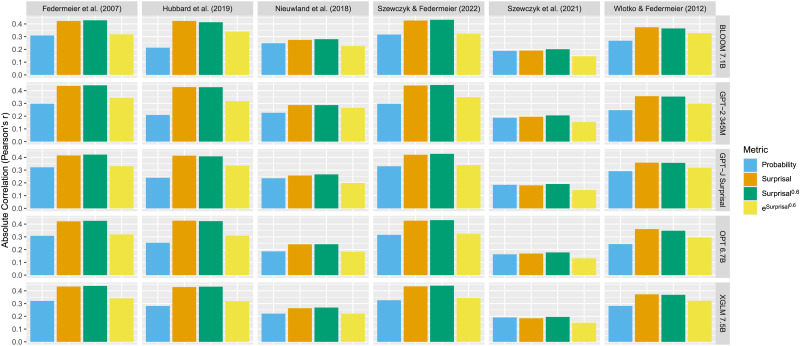
The absolute correlation coefficient between the probability, surprisal, surprisal^0.6^, and *e*^surprisal^0.6^^ calculated from each language model and contextual similarity.

Second, we test how closely contextual similarity correlates with each of the same model-derived metrics. We show the results in Figure 6. Again, we begin by considering how well these results align with our formulation of Szewczyk and Federmeier’s (2022) multiple sub-component account. Under this account, we would expect surprisal to correlate more closely with contextual similarity than probability does. This is the case for 28 of the total 30 comparisons. Taken together with the previous results, this supports the idea that language model probability better captures lexical prediction (operationalized by cloze probability) than surprisal, and surprisal (to a greater extent) better captures contextual similarity.

However, it is also worth noting that surprisal^0.6^ is always more closely correlated with contextual similarity than probability, and is more closely correlated than surprisal in 20 of the 30 comparisons.

### Discussion

The results of this analysis are mixed. The fact that in almost all (28 out of 30) comparisons, language model surprisal is more strongly correlated with contextual similarity than probability supports Szewczyk and Federmeier’s (2022) claim that surprisal may operationalize the semantic featural overlap between a critical word and its context. The fact that language model probability is more strongly correlated with cloze probability in 19 out of 30 comparisons also supports the account, though less strongly.

We also see that surprisal^0.6^ is generally the variable most strongly correlated with cloze probability, while *e*^surprisal^0.6^^ shows the weakest correlation. Thus, our results suggest if there is a sublogarithmic relationship between statistical probability and the N400, the sublogarithmic relationship lies between statistical and subjective probability. For this reason, they also provide indirect evidence for the contextual probability account in general, as the same mathematical relationship best characterizes both the relationship between language model probability and cloze probability and between language model probability and N400 amplitude.

Finally, we consider the extent to which these results shed light on the question of whether the multiple sub-component account or the sublogarithmic account better explains the data. On most of the models and datasets, we see the same patterns. First, we see that language model surprisal tends to be more correlated with contextual similarity than language model probability is. In addition, we see that language model probability tends to be more correlated with cloze probability than language model surprisal is. These findings are both in line with what we would expect to see under the multiple sub-component account. However, the evidential support for the account is somewhat undercut by the fact that surprisal^0.6^ is a better predictor of both. Thus, the results provide some evidence in favor of each possible account, without providing clear support for one over the other.

The fact that surprisal^0.6^ is the best predictor of both our metric of semantic feature overlap (contetxual similarity) and lexical prediction (cloze probability) suggests that cloze probability may not clearly reflect lexical prediction as distinct from semantic feature overlap. This is further supported by previous work showing a correlation between contextual similarity and cloze probability (Michaelov et al., 2024) as well as between word association and cloze probability (Smith & Levy, 2011). Indeed, in the datasets we analyze, cloze probability and contextual similarity are generally weakly to moderately correlated (Federmeier et al. (2007): *r* = 0.344; Hubbard et al. (2019): *r* = 0.294; Nieuwland et al. (2018): *r* = 0.244; Szewczyk and Federmeier (2022): *r* = 0.343; Szewczyk et al. (2022): *r* = 0.179; Wlotko and Federmeier (2012): *r* = 0.301). Whether this has implications for the extent to which semantic feature overlap and lexical prediction are dissociable in general is a question for future work.

## GENERAL DISCUSSION

The work described in this paper is novel in several ways. To the best of our knowledge, it is the first to compare linear, logarithmic, super-logarithmic, and sub-logarithmic relationships between probability and N400 amplitude in explaining experimental data. The key and surprising finding is that on the whole, sub-linearly-transformed surprisal appears to be a better predictor of N400 amplitude than surprisal is. We found that on three of the six datasets tested, predicting N400 amplitude using sub-linearly-transformed surprisal leads to regressions with a substantially better fit than using un-transformed surprisal; while the reverse pattern was only seen on one of the datasets. Moreover, we observed that when calculating the metrics using the language models that best predict N400 amplitude for each dataset, sub-linearly-transformed surprisal almost always explains all the variance explained by surprisal and probability. This is in contrast to previous work on reading time, where to date there is no clear consensus on whether a linear, sub-linear, or super-linear relationship with surprisal best explains the data (Meister et al., 2021; Shain et al., 2024; Smith & Levy, 2013; Wilcox et al., 2023).

Beyond this new finding, the current results also replicate and expand upon previous work. First, in line with Yan and Jaeger (2020) and Szewczyk and Federmeier (2022), surprisal out-performed un-transformed probability as a single predictor of N400 amplitude—across language models, using surprisal as a predictor results in significantly better-fitting linear mixed-effects models than probability. We show that this is not just the case for the Frank and Willems (2017) model used by Yan and Jaeger (2020) and for the GPT-2 (Radford et al., 2019) model used by Szewczyk and Federmeier (2022), but that it occurs generally across contemporary autoregressive transformer language models. While there are individual exceptions, overall across datasets, language model surprisal out-performs un-transformed probability.

The results reported here also further support the finding, reported by Szewczyk and Federmeier (2022), that language model probability can explain variance in N400 amplitude above and beyond that explained by surprisal. Szewczyk and Federmeier (2022) found this using GPT-2 1.5B; our results similarly show that for the Federmeier et al. (2007) and Hubbard et al. (2019) datasets, GPT-J 6B, GPT-23 45M, OPT 6.7B, and XGLM 7.5B probability explain variance in N400 not explained by the surprisal of the same language model; and the same is true for BLOOM 7.1B on the Federmeier et al. (2007) dataset. This suggests that the significant effect of probability (above and beyond surprisal) on N400 amplitude is not due to possible idiosyncrasies of GPT-2 1.5B, but may instead reflect a more general trend. As previously noted, however, the additional variance explained by probability can generally also be accounted for by a sub-linear transformation of surprisal.

It is worth briefly noting that previous work has generally shown that language models that are trained on more data and that are better at natural language processing tasks overall also tend to perform best at predicting N400 amplitude (Aurnhammer & Frank, 2019a, 2019b; Frank et al., 2015; Merkx & Frank, 2021; Michaelov & Bergen, 2020; Michaelov et al., 2021, 2022). On the other hand, several recent studies have suggested that increasing model scale past a certain point leads to decreased performance in modeling reading time (de Varda & Marelli, 2023; Oh & Schuler, 2023; Oh et al., 2022; Shain et al., 2024). The results of the present study are more in line with the former set of findings—the models that best predict N400 amplitude on five out of six datasets (GPT-J 6B, OPT 6.7B, BLOOM 7.1B, XGLM 7.5B) are among the largest we tested, both in terms of parameter count (6–7.5 billion parameters) and training data size (180 billion to 500 billion tokens).

### Theoretical Implications

The results show that the single best predictor of N400 amplitude across all datasets is surprisal^0.6^, that surprisal is generally a better predictor than un-transformed probability, and that, in line with the results of Szewczyk and Federmeier (2022), probability can explain variance in N400 amplitude not explained by surprisal. Overall, the empirical results are most consistent with either the multiple sub-component account of Szewczyk and Federmeier (2022) where there is both a linear and logarithmic relationship between statistical probability and N400 amplitude, or with a sub-logarithmic variant of the proportional preactivation account, where there is a sub-logarithmic relationship between statistical probability and the extent to which words are preactivated, with a linear relationship holding between this preactivation and the N400.

However, there is no clear single relationship suggested by the study. As noted, no set of metrics derived from language model probability consistently predict N400 amplitude better than others in all cases. While surprisal^0.6^ is the best single predictor of N400 amplitude, the probability and surprisal calculated by some language models can explain each additional variance on one of the datasets (Nieuwland et al., 2018).

We also see a similar lack of clarity when we compare how well different transformations of language model probability correlate with metrics thought to correlate with the factors that Szewczyk and Federmeier (2022) argue play important, dissociable roles in the neurocognitive processes underlying the N400—contextual similarity, which has been argued to model semantic feature overlap (Michaelov et al., 2024), and cloze probability, which can be used to model lexical prediction (Brothers & Kuperberg, 2021). Language model surprisal correlates more closely with the cosine similarity between the embeddings of critical words and their contexts than language model probability does, suggesting that it better reflects semantic feature overlap, in line with the account of Szewczyk and Federmeier (2022). However, we also find that in the majority of cases, surprisal^0.6^ is more correlated with this metric of semantic feature overlap than is surprisal, which presents a problem for the account. Similarly, language model probability is more closely correlated than language model suprisal with cloze probability, a metric of lexical prediction, in the majority of cases, which is also in line with the account of Szewczyk and Federmeier (2022). Again, however, surprisal^0.6^ is more closely correlated with cloze probability—in fact, this is so for all comparisons of language models and datasets carried out. The fact that surprisal^0.6^ is more closely correlated with cloze probability and contextual similarity than the other metrics may be taken to suggest that the latter two metrics are correlated, and indeed we find that they are, but to less of an extent than surprisal^0.6^ is to either.

Taken together, the results defy a single straightforward conclusion as to the mathematical relationship between statistical probability and N400 amplitude. However, we believe that the present study has brought us closer to characterizing this relationship by identifying several of its features. First, the relationship between statistical probability and N400 amplitude does not appear to be a simple linear or logarithmic one. Second, the results suggest that the N400 is more sensitive to differences at the lower end of the scale than can captured by probability (and thus closer to those reflected in surprisal or surprisal^0.6^), but also more sensitive to the differences at the higher end of the probability scale than can be captured by surprisal (and thus closer to those reflected in probability or surprisal^0.6^). Finally, in our sample at least, we see that the metric that best predicts N400 amplitude alone is one that reflects both contextual predictability (as operationalized by cloze) and semantic feature overlap (as operationalized by contextual similarity; see also Michaelov et al., 2021).

This last finding is additionally worth highlighting because in virtually all previous work suggesting a nonlinear relationship between language model probability and processing difficulty, the nonlinearity is proposed to arise based on how subjective probabilities lead to differences in processing difficulty. Our results, however, suggest that the nonlinearity—which we find to sub-logarithmic—instead lies between statistical probabilities (as operationalized by language model probabilities) and these subjective probabilities.

## CONCLUSIONS

In this study, we set out to compare how well linear, logarithmic, and exponentiated (i.e., super- and sub-) logarithmic transformations of contextual probability correlate with the N400, a neural index of processing difficulty. In line with previous work (Szewczyk & Federmeier, 2022; Yan & Jaeger, 2020), we find that that surprisal, a logarithmic transformation of probability, out-performs probability as a predictor of N400 amplitude. However, as has previously been reported (Szewczyk & Federmeier, 2022), we find that probability can explain variance in N400 amplitude not explained by surprisal.

Our novel finding, and one that lies in contrast to previous work on reading time as an index of processing difficulty (Meister et al., 2021; Shain et al., 2024; Smith & Levy, 2013; Wilcox et al., 2023), is that sub-logarithmically transformed probability is a better predictor of N400 amplitude than surprisal. Specifically, we find for almost all language models and datasets, surprisal^0.6^ explains at least as much variance in N400 amplitude as both surprisal and probability; suggesting that the relationship between probability and N400 amplitude may in fact be sub-logarithmic.

The fact that this result is not accounted for by any previous work highlights the importance of not viewing language processing as a monolith—different metrics of contextual probability (cloze vs. language model) and different metrics of processing difficulty (reading time vs. the N400) show distinct patterns; and those different patterns may ultimately be key to understanding the processes of language comprehension.

## ACKNOWLEDGMENTS

We would like to thank the members and affiliates of the Language and Cognition Lab at UCSD for their valuable discussion, and the editor and anonymous reviewers for their helpful feedback. All language models were run on hardware provided by the NVIDIA Corporation as part of an NVIDIA Academic Hardware Grant.

## AUTHOR CONTRIBUTIONS

JM: Conceptualization, Data curation, Formal analysis, Investigation, Methodology, Software, Visualization, Writing – original draft, Writing – review & editing; BB: Conceptualization, Supervision, Writing – review & editing.

## DATA AVAILABILITY STATEMENT

We provide all the code, data, and statistical analysis scripts required to reproduce our results at https://osf.io/w5hez.

## Note

^1^ In the Appendix to the paper, available at https://www.researchgate.net/publication/221618546.

## Figures and Tables

**Figure B1.**
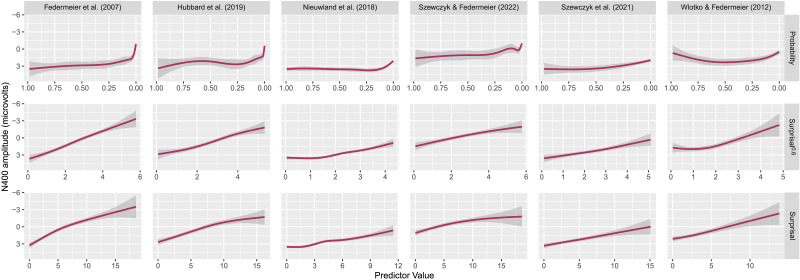
N400 amplitude as a function of BLOOM 7.1B probability, surprisal, and surprisal^0.6^. The x-axis for probability is reversed for easier comparison with surprisal and surprisal^0.6^.

**Figure B2.**
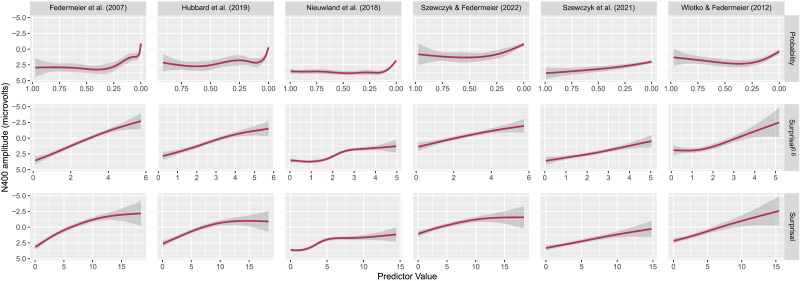
N400 amplitude as a function of OPT 6.7B probability, surprisal, and surprisal^0.6^. The x-axis for probability is reversed for easier comparison with surprisal and surprisal^0.6^.

**Figure B3.**
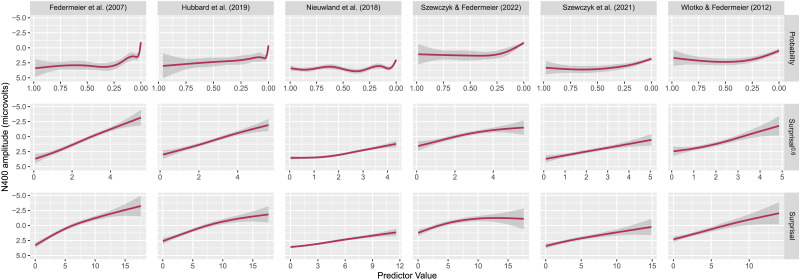
N400 amplitude as a function of XGLM 7.5B probability, surprisal, and surprisal^0.6^. The x-axis for probability is reversed for easier comparison with surprisal and surprisal^0.6^.

**Figure B4.**
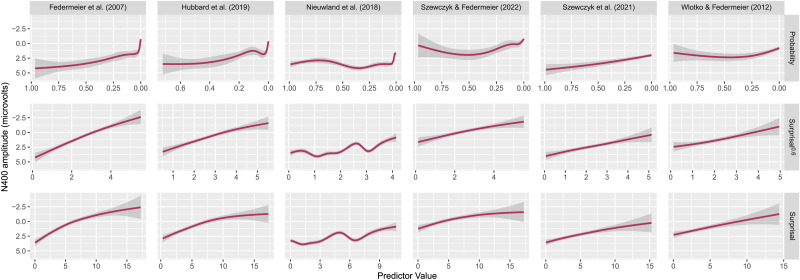
N400 amplitude as a function of GPT-2 345M probability, surprisal, and surprisal^0.6^. The x-axis for probability is reversed for easier comparison with surprisal and surprisal^0.6^.

## APPENDIX A

### Statistical Analysis of Regression Fit (Analysis 2)

In this appendix, we include the supplementary statistical analyses investigating whether linear mixed-effects regression models with probability, surprisal, or surprisal^0.6^ as predictors are best able to predict N400 amplitude. We compare the AIC of regressions with probability or surprisal as predictors (Table A1), probability or surprisal^0.6^ as predictors (Table A2), and surprisal or surprisal^0.6^ as predictors (Table A3).

**Table A1.** Results of likelihood ratio tests and coefficients of linear mixed-effects models testing the difference in the AIC of regressions using probability or surprisal as a predictor of N400 amplitude on each dataset.

**Likelihood ratio test**			**Coefficient**		
**Dataset**	***χ*^2^(1)**	***p***	**Estimate**	**SE**	**t**
Nieuwland et al. (2018)	70.64	<0.0001	−41.91	2.87	−14.58
Federmeier et al. (2007)	109.23	<0.0001	−51.85	2.00	−25.91
Wlotko and Federmeier (2012)	95.82	<0.0001	−22.44	1.05	−21.35
Szewczyk and Federmeier (2022)	27.79	<0.0001	−5.72	0.89	−6.43
Hubbard et al. (2019)	62.70	<0.0001	−18.23	1.42	−12.82
Szewczyk et al. (2022)	76.75	<0.0001	−11.89	0.74	−16.05

**Table A2.** Results of likelihood ratio tests and coefficients of linear mixed-effects regression models testing the difference in the AIC of regressions using probability or surprisal^0.6^ as a predictor of N400 amplitude on each dataset.

**Likelihood ratio test**			**Coefficient**		
**Dataset**	***χ*^2^(1)**	***p***	**Estimate**	**SE**	**t**
Nieuwland et al. (2018)	92.46	<0.0001	−37.53	1.85	−20.33
Federmeier et al. (2007)	137.87	<0.0001	−66.15	1.71	−38.69
Wlotko and Federmeier (2012)	110.11	<0.0001	−20.05	0.76	−26.24
Szewczyk and Federmeier (2022)	78.73	<0.0001	−10.86	0.66	−16.54
Hubbard et al. (2019)	106.17	<0.0001	−24.43	0.98	−25.47
Szewczyk et al. (2022)	108.02	<0.0001	−13.41	0.53	−25.47

**Table A3.** Results of likelihood ratio tests and coefficients of linear mixed-effects models testing the difference in the AIC of regressions using surprisal or surprisal^0.6^ as a predictor of N400 amplitude on each dataset.

**Likelihood ratio test**			**Coefficient**		
**Dataset**	***χ*^2^(1)**	***p***	**Estimate**	**SE**	**t**
Nieuwland et al. (2018)	13.61	0.0049	4.38	1.08	4.06
Federmeier et al. (2007)	124.42	<0.0001	−14.30	0.45	−32.11
Wlotko and Federmeier (2012)	33.04	<0.0001	2.39	0.33	7.30
Szewczyk and Federmeier (2022)	85.82	<0.0001	−5.14	0.28	−18.42
Hubbard et al. (2019)	53.33	<0.0001	−6.19	0.57	−10.93
Szewczyk et al. (2022)	28.03	<0.0001	−1.52	0.23	−6.48

## APPENDIX B

### Language Model Probability and N400 Amplitude Plots (Analysis 3)

In this appendix, we plot the relationship between probability, surprisal, or surprisal^0.6^ and N400 amplitude, with probabilities calculated using BLOOM 7.1B (Figure B1), OPT 6.7B (Figure B2), XGLM 7.5B (Figure B3), and GPT-2 345M (Figure B4). The plot for GPT-J 6B is included in the main body of the paper (Figure 3).

## APPENDIX C

### Comparison of Regression AICs Including Probability (Analysis 3)

In Analysis 3, we compare the fit of regressions of including probability, surprisal, surprisal^0.6^, and combinations of these metrics as predictors of N400 amplitude. Because regressions with probability as a predictor (and not also either surprisal or surprisal^0.6^) often perform far worse than the others, we do not include them in Figure 4 in the main body of the paper to increase the ease of comparing the performance of the other regressions. For completeness, we provide the full comparison in Figure C1.

## APPENDIX D

### Individual Language Model Statistical Analyses (Analysis 3)

In this appendix, we include the statistical analyses investigating the whether adding probability (*p*), surprisal (*S*), surprisal^0.6^ (*S*^0.6^), or a combination of these to a linear mixed-effects model already including one or more other of these variables as predictors significantly improves model fit. In this way, we test which of these metrics explain variance in N400 amplitude not explained by the others. We provide the results for the metrics calculated using BLOOM 7.1B (Table D1), OPT 6.7B (Table D2), XGLM 7.5B (Table D3), and GPT-2 345M (Table D4). The table for GPT-J 6B is included in the main body of the paper (Table 3).

**Table D1.** Results of the likelihood ratio tests testing the effect of adding BLOOM 7.1B probability (*p*), surprisal (*S*), surprisal^0.6^ (*S*^0.6^), or a combination of these to a linear mixed-effects model already including one or more other of these variables, thereby testing whether they explain any additional variance. As an example, *S* + *P* refers to a likelihood ratio test of whether probability explains additional variance in N400 amplitude above and beyond that explained by surprisal. FWOK07 refers to Federmeier et al. (2007), WF12 to Wlotko and Federmeier (2012), SF22 to Szewczyk and Federmeier (2022), HRJF19 to Hubbard et al. (2019), SMF22 to Szewczyk et al. (2022), and N18 to Nieuwland et al. (2018).

**Dataset**	***S*^0.6^ + *S***		***S*^0.6^ + *P***		***S* + *S*^0.6^**		***S* + *P***	
	***χ*^2^(1)**	***p***	***χ*^2^(1)**	***p***	***χ*^2^(1)**	***p***	***χ*^2^(1)**	***p***
FWOK07	0.00	1.0000	0.01	1.0000	**10.46**	**0.0203**	**10.15**	**0.0232**
WF12	6.96	0.1159	6.90	0.1186	0.94	1.0000	0.80	1.0000
SF22	0.91	1.0000	0.31	1.0000	6.25	0.1652	5.48	0.2419
HRJF19	0.41	1.0000	0.00	1.0000	7.21	0.1024	5.69	0.2187
SMF22	0.26	1.0000	0.00	1.0000	0.84	1.0000	1.49	1.0000
N18	**10.40**	**0.0205**	**10.74**	**0.0182**	1.47	1.0000	1.20	1.0000
**Dataset**	***P* + *S***		***P* + *S*^0.6^**		**(*S* + *P*) + *S*^0.6^**		***S*^0.6^ + (*S* + *P*)**	
***χ*^2^(1)**	***p***	***χ*^2^(1)**	***p***	***χ*^2^(1)**	***p***	***χ*^2^(2)**	***p***
FWOK07	**76.01**	**<0.0001**	**76.32**	**<0.0001**	0.32	1.0000	0.02	1.0000
WF12	**32.69**	**<0.0001**	**32.77**	**<0.0001**	0.31	1.0000	7.13	0.3327
SF22	**12.09**	**0.0104**	**12.25**	**0.0097**	0.97	1.0000	1.11	1.0000
HRJF19	**28.11**	**<0.0001**	**29.23**	**<0.0001**	2.77	0.9825	1.65	1.0000
SMF22	**15.51**	**0.0021**	**14.6**	**0.0031**	2.92	0.9155	3.83	1.0000
N18	**47.67**	**<0.0001**	**48.28**	**<0.0001**	0.68	1.0000	10.81	0.0672

**Table D2.** Results of the likelihood ratio tests testing the effect of adding OPT 6.7B probability (*P*), surprisal (*S*), surprisal^0.6^ (*S*^0.6^), or a combination of these to a linear mixed-effects model already including one or more other of these variables, thereby testing whether they explain any additional variance. As an example, *S* + *P* refers to a likelihood ratio test of whether probability explains additional variance in N400 amplitude above and beyond that explained by surprisal. FWOK07 refers to Federmeier et al. (2007), WF12 to Wlotko and Federmeier (2012), SF22 to Szewczyk and Federmeier (2022), HRJF19 to Hubbard et al. (2019), SMF22 to Szewczyk et al. (2022), and N18 to Nieuwland et al. (2018).

**Dataset**	***S*^0.6^ + *S***		***S*^0.6^ + *P***		***S* + *S*^0.6^**		***S* + *P***	
	***χ*^2^(1)**	***p***	***χ*^2^(1)**	***p***	***χ*^2^(1)**	***p***	***χ*^2^(1)**	***p***
FWOK07	0.51	1.0000	0.60	1.0000	**14.45**	**0.0033**	**14.93**	**0.0027**
WF12	5.46	0.2435	7.61	0.0837	0.41	1.0000	0.76	1.0000
SF22	0.82	1.0000	0.33	1.0000	5.69	0.2187	5.14	0.2817
HRJF19	4.04	0.5019	0.92	1.0000	**15.46**	**0.0021**	**11.40**	**0.0137**
SMF22	0.75	1.0000	0.52	1.0000	0.23	1.0000	0.19	1.0000
N18	2.80	0.9702	8.30	0.0602	0.49	1.0000	0.10	1.0000
**Dataset**	***P* + *S***		***P* + *S*^0.6^**		**(*S* + *P*) + *S*^0.6^**		***S*^0.6^ + (*S* + *P*)**	
	***χ*^2^(1)**	***p***	***χ*^2^(1)**	***p***	***χ*^2^(1)**	***p***	***χ*^2^(2)**	***p***
FWOK07	**59.01**	**<0.0001**	**58.62**	**<0.0001**	0.02	1.0000	1.00	1.0000
WF12	**33.05**	**<0.0001**	**34.85**	**<0.0001**	1.95	1.0000	7.76	0.2542
SF22	**11.07**	**0.0157**	**11.13**	**0.0155**	0.64	1.0000	0.91	1.0000
HRJF19	**16.11**	**0.0015**	**17.05**	**0.0010**	7.81	0.0768	7.79	0.2522
SMF22	**18.15**	**0.0006**	**17.95**	**0.0006**	0.07	1.0000	0.78	1.0000
N18	**39.10**	**<0.0001**	**44.99**	**<0.0001**	4.88	0.3223	7.29	0.3126

**Table D3.** Results of the likelihood ratio tests testing the effect of adding XGLM 7.5B probability (*P*), surprisal (*S*), surprisal^0.6^ (*S*^0.6^), or a combination of these to a linear mixed-effects model already including one or more other of these variables, thereby testing whether they explain any additional variance. As an example, *S* + *P* refers to a likelihood ratio test of whether probability explains additional variance in N400 amplitude above and beyond that explained by surprisal. FWOK07 refers to Federmeier et al. (2007), WF12 to Wlotko and Federmeier (2012), SF22 to Szewczyk and Federmeier (2022), HRJF19 to Hubbard et al. (2019), SMF22 to Szewczyk et al. (2022), and N18 to Nieuwland et al. (2018).

**Dataset**	***S*^0.6^ + *S***		***S*^0.6^ + *P***		***S* + *S*^0.6^**		***S* + *P***	
	***χ*^2^(1)**	***p***	***χ*^2^(1)**	***p***	***χ*^2^(1)**	***p***	***χ*^2^(1)**	***p***
FWOK07	0.01	1.0000	0.01	1.0000	**11.44**	**0.0136**	**11.55**	**0.0131**
WF12	0.61	1.0000	1.18	1.0000	0.91	1.0000	0.60	1.0000
SF22	3.47	0.6804	1.18	1.0000	**11.09**	**0.0156**	**9.42**	**0.0332**
HRJF19	2.94	0.912	0.99	1.0000	**13.33**	**0.0057**	**10.56**	**0.0194**
SMF22	0.01	1.0000	0.15	1.0000	1.82	1.0000	2.81	0.9699
N18	8.23	0.062	**9.90**	**0.0258**	0.61	1.0000	0.97	1.0000
**Dataset**	***P* + *S***		***P* + *S*^0.6^**		**(*S* + *P*) + *S*^0.6^**		***S*^0.6^ + (*S* + *P*)**	
	***χ*^2^(1)**	***p***	***χ*^2^(1)**	***p***	***χ*^2^(1)**	***p***	***χ*^2^(2)**	***p***
FWOK07	**79.34**	**<0.0001**	**79.23**	**<0.0001**	0.08	1.0000	0.20	1.0000
WF12	**20.69**	**0.0002**	**21.56**	**0.0001**	1.53	1.0000	1.83	1.0000
SF22	7.38	0.0941	6.76	0.1270	2.36	1.0000	4.16	1.0000
HRJF19	**18.39**	**0.0005**	**19.21**	**0.0003**	4.33	0.4323	4.50	1.0000
SMF22	**12.50**	**0.0087**	**11.66**	**0.0125**	3.19	0.7922	4.19	1.0000
N18	**50.80**	**<0.0001**	**52.11**	**<0.0001**	1.70	1.0000	10.29	0.0837

**Table D4.** Results of the likelihood ratio tests testing the effect of adding GPT-2 345M probability (*P*), surprisal (*S*), surprisal^0.6^ (*S*^0.6^), or a combination of these to a linear mixed-effects model already including one or more other of these variables, thereby testing whether they explain any additional variance. As an example, *S* + *P* refers to a likelihood ratio test of whether probability explains additional variance in N400 amplitude above and beyond that explained by surprisal. FWOK07 refers to Federmeier et al. (2007), WF12 to Wlotko and Federmeier (2012), SF22 to Szewczyk and Federmeier (2022), HRJF19 to Hubbard et al. (2019), SMF22 to Szewczyk et al. (2022), and N18 to Nieuwland et al. (2018).

**Dataset**	***S*^0.6^ + *S***		***S*^0.6^ + *P***		***S* + *S*^0.6^**		***S* + *P***	
	***χ*^2^(1)**	***p***	***χ*^2^(1)**	***p***	***χ*^2^(1)**	***p***	***χ*^2^(1)**	***p***
FWOK07	3.63	0.6258	3.46	0.6804	**21.26**	**0.0001**	**20.24**	**0.0002**
WF12	0.21	1.0000	0.27	1.0000	0.82	1.0000	0.91	1.0000
SF22	1.47	1.0000	0.49	1.0000	6.61	0.1375	6.00	0.1858
HRJF19	5.20	0.2741	2.30	1.0000	**14.67**	**0.0030**	**11.21**	**0.0150**
SMF22	0.14	1.0000	0.60	1.0000	3.50	0.6757	4.10	0.4891
N18	3.74	0.5907	4.58	0.3779	0.01	1.000	0.00	1.0000
**Dataset**	***P* + *S***		***P* + *S*^0.6^**		**(*S* + *P*) + *S*^0.6^**		***S*^0.6^ + (*S* + *P*)**	
	***χ*^2^(1)**	***p***	***χ*^2^(1)**	***p***	***χ*^2^(1)**	***p***	***χ*^2^(2)**	***p***
FWOK07	**45.84**	**<0.0001**	**46.69**	**<0.0001**	1.03	1.0000	3.64	1.0000
WF12	**14.37**	**0.0034**	**14.34**	**0.0034**	0.04	1.0000	0.34	1.0000
SF22	**10.44**	**0.0203**	**10.06**	**0.0241**	0.64	1.0000	1.50	1.0000
HRJF19	**15.33**	**0.0022**	**15.90**	**0.0017**	4.55	0.3812	6.29	0.4891
SMF22	**11.67**	**0.0125**	**11.52**	**0.0131**	0.58	1.0000	1.33	1.0000
N18	**43.30**	**<0.0001**	**44.14**	**<0.0001**	0.26	1.0000	4.00	1.0000
